# The time-course of real-world scene perception: Spatial and semantic processing

**DOI:** 10.1016/j.isci.2022.105633

**Published:** 2022-11-19

**Authors:** Matt D. Anderson, James H. Elder, Erich W. Graf, Wendy J. Adams

**Affiliations:** 1 Centre for Perception and Cognition, Psychology, University of Southampton, Southampton, UK; 2 Centre for Vision Research, Department of Psychology, Department of Electrical Engineering and Computer Science, York University, Toronto, Canada

**Keywords:** Biological sciences, Neuroscience, Sensory neuroscience

## Abstract

Real-world scene perception unfolds remarkably quickly, yet the underlying visual processes are poorly understood. Space-centered theory maintains that a scene’s spatial structure (e.g., openness, mean depth) can be rapidly recovered from low-level image statistics. In turn, the statistical relationship between a scene’s spatial properties and semantic content allows for semantic identity to be inferred from its layout. We tested this theory by investigating (1) the temporal dynamics of spatial and semantic perception in real-world scenes, and (2) dependencies between spatial and semantic judgments. Participants viewed backward-masked images for 13.3 to 106.7 ms, and identified the semantic (e.g., beach, road) or spatial structure (e.g., open, closed-off) category. We found no temporal precedence of spatial discrimination relative to semantic discrimination. Computational analyses further suggest that, instead of using spatial layout to infer semantic categories, humans exploit semantic information to discriminate spatial structure categories. These findings challenge traditional ‘bottom-up’ views of scene perception.

## Highlights

• Spatial structure and semantic perception share a similar time-course
• Humans infer spatial structure from semantics shortly after image onset
• Yet, humans do not reliably infer semantics from spatial structure

## Graphical abstract

**Figure:**
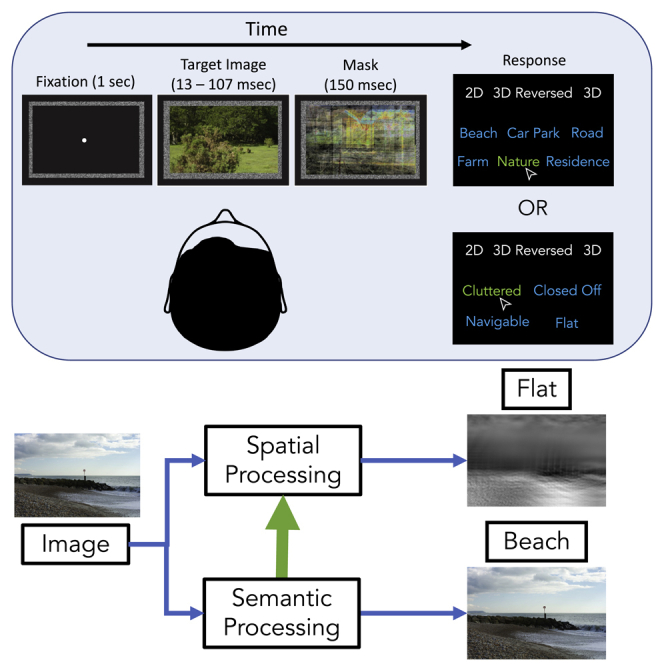

## Introduction

Efficient scene recognition is a hallmark of human visual processing. Humans can accurately discriminate different semantic scene categories (e.g., beach, forest, and street)^1^^,^^2^ after viewing an image for less than 20 ms – a fraction of the duration of a single eye fixation. Despite this, initial representations of low-level image features are noisy and imprecise, as visual information is acquired over time.^3^^,^^4^

**Figure 1:**
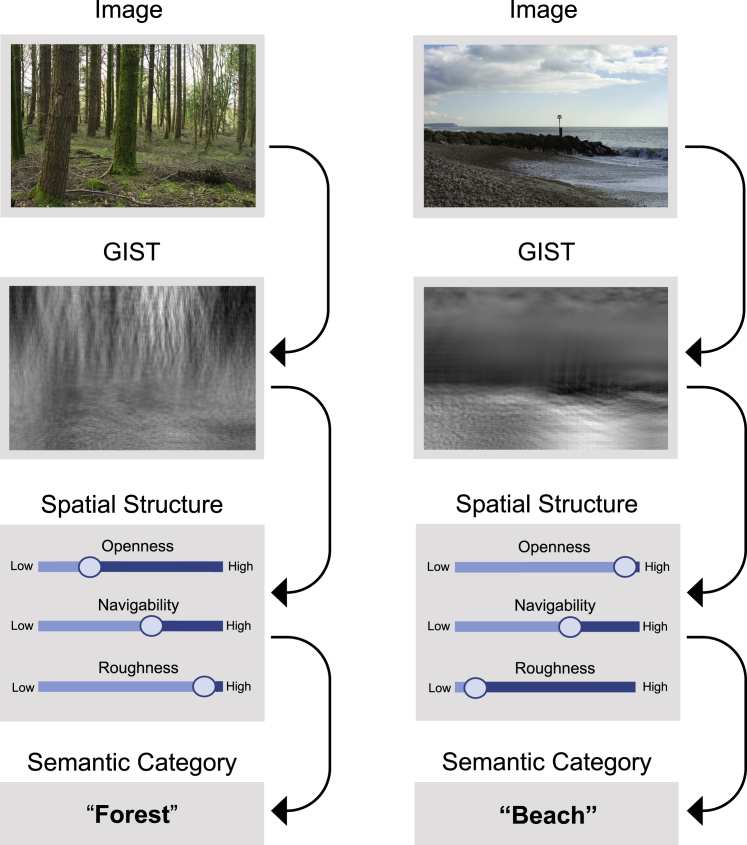
The space-centered theory of real-world scene perception Shortly after image onset, humans encode low-dimensional GIST statistics. GIST images (second row) visualize the information lost/preserved by GIST transformation. To produce these images, white noise images were iteratively coerced to have the same GIST features as the original image.^58^ The ‘textured’ appearance reflects the energy distribution across different orientations and spatial frequencies. Humans are thought to use image GIST to estimate spatial structure properties such as the degree of openness, navigability, and roughness.^5^^,^^6^^,^^7^ In turn, these properties predict the semantic category of the image. This route to real-world scene perception is thought to bypass computationally expensive object segmentation processes.^5^^,^^6^^,^^7^ Example images are drawn from the SYNS database.^55^

The space-centered theory^5^^,^^6^^,^^7^ (see Figure 1) has had a tremendous influence on current understanding of real-world scene perception.^8^ According to space-centered theory, semantic category is inferred from a scene’s three-dimensional structural properties (e.g., ‘Openness’, ‘Navigability’, ‘Mean Depth’). These structural properties are, in turn, estimated from low-level, global summary statistics that can be efficiently encoded from complex images. The widely-cited GIST summary statistic encodes two-dimensional image energy over a small number of orientations and spatial scales.^5^ Studies suggest that GIST representations are diagnostic of three-dimensional spatial structure. For example, images rated high in openness (e.g., beaches and countrysides) contain relatively more high-contrast horizontal edges, whereas images low in openness (e.g., forests and city centers) contain more vertical edges.^5^ Early image processing is biased toward global image features,^9^^,^^10^^,^^11^ whereas local image features corresponding to individual objects/elements, are processed later.^9^^,^^10^^,^^11^ Thus, a central appeal of space-centered theory is that summary statistics might provide a fast route to semantic categorization,^5^^,^^12^^,^^13^ without the need to extract phase-dependent, local information required for object recognition.

If humans encode spatial structure to discriminate scene semantics, spatial structure properties should be perceptually available *before* semantics. One way to explore this prediction is to manipulate the duration of the stimulus, using an effective backward mask to limit later processing and feedback, and then assess how semantic and spatial category perception evolve with time. This approach has generated insights into the neural processes underlying perceptual grouping,^14^ object and scene recognition,^3^ and spatial frequency processing.^15^ For scene processing, Greene and Oliva^2^ found that the minimum presentation time required to discriminate global scene properties (like openness, mean depth, navigability, temperature, and naturalness) at 75% accuracy was significantly shorter than the minimum time required for semantic discrimination (34 versus 50 ms). Although this finding seems to support the space-centered theory, there are several limitations to this study. First, scene naturalness is treated as a spatial structure dimension (as is common in this literature^6^), but it could equally describe a semantic category system (natural versus man-made). Furthermore, Greene and Oliva found that several individual spatial structure dimensions actually had *longer* minimum presentation times (openness: 47 ms, navigability: 36 ms) than some basic-level semantic categories (mountain: 46 ms, forest: 30 ms).

A second test of the space-centered theory is whether human semantic categorization responses are predicted by diagnostic spatial structure image features. Greene and Oliva^1^ presented observers with images of real-world scenes for 30 ms, and compared semantic category responses with those of a model trained to predict semantic category from spatial structure dimensions. They found a strong correlation between human and model categorization for accuracy-defined bins, but did not compare human and model responses at the level of individual images. Other studies confer only partial support. Adaptation to images high in openness (e.g., ocean, desert, canyon) biases the categorizing of novel stimuli toward low-openness categories (e.g., fields categorized as forests), but adaptation to *low*-openness images does not generate a corresponding bias toward high-openness categories (e.g., forests categorized as fields).^16^ The cause of this asymmetry is unclear, but may suggest that these property dimensions are poorly operationalized, that they are weakly relied on to discriminate semantic categories, or that they themselves resemble categories rather than continuous dimensions (among other explanations).

Evidence for the assumption that semantic categorization is primarily driven by global image features is also limited. GIST features are unreliable predictors of spatial structure properties across different image databases.^17^ Moreover, objects are processed just as quickly as entire scenes,^18^^,^^19^^,^^20^^,^^21^ and scene categorization is impaired when embedded objects are incongruent with the scene (e.g., a man-made object in a natural scene).^19^^,^^22^^,^^23^^,^^24^ Neuroimaging studies also suggest that scene category representations can be decoded from object co-occurrence statistics,^25^ and that semantically congruent objects improve decoding accuracy – particularly under conditions of image degradation.^26^ These results suggest that scene recognition relies on local object processing.

In summary, although the space-centered theory of scene category perception continues to be influential, empirical support is mixed. Here we get at the heart of the matter by explicitly testing two core predictions of space-centered theory that have thus far received limited attention: (1) That spatial structure discrimination precedes semantic discrimination, and (2) that semantic discrimination is *informed by* spatial structure properties.

**Figure 2:**
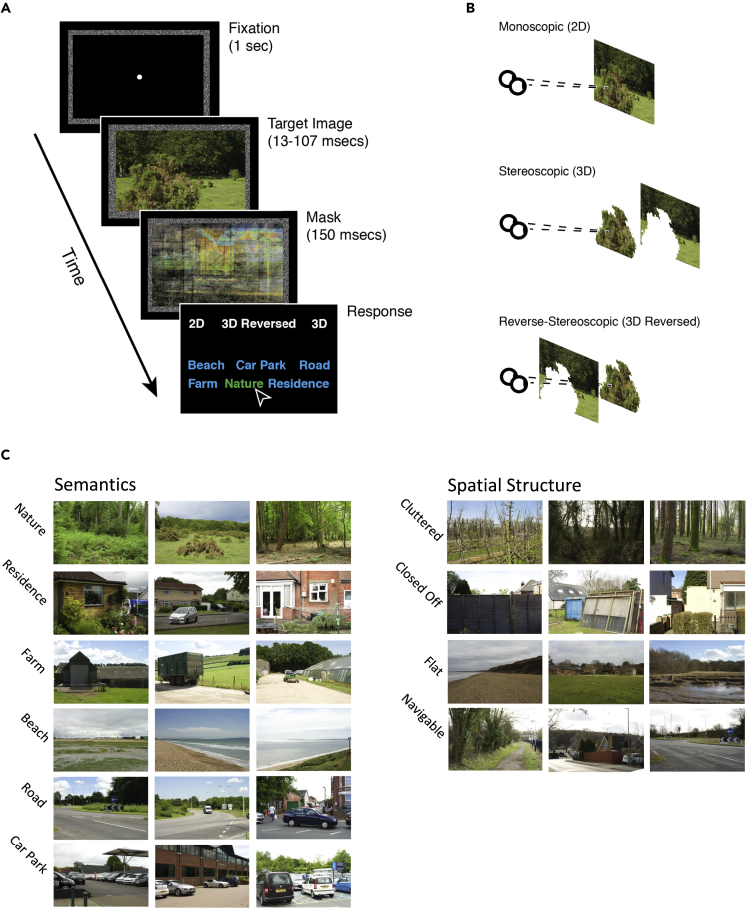
Stimuli and procedure (A) Semantic task procedure. Participants reported the category of the target image and the binocular viewing condition. The spatial structure task procedure was the same but with different category labels. (B) Schematic of the different binocular disparity conditions. In the monoscopic condition, both eyes see the same image. In the stereoscopic condition, both eyes see corresponding stereo pairs. In the reverse-stereo condition, left and right images are swapped. Note that although the reverse-stereo condition inverts the depth, the locations of boundaries signaled by depth discontinuities are preserved. (C) The semantic (left) and spatial structure (right) categories, with example images. Stimuli were drawn from the SYNS database.^55^

Part 1 of this paper reports the results of an experimental study that tests the first prediction. Participants viewed briefly-presented images of real-world scenes (Figure 2A). We measured categorization responses as a function of presentation duration (13, 27, 53, 107 ms), and the availability of color and binocular disparity cues. Participants identified the semantic category (road, farm, nature, beach, car park, residence) or spatial structure category (flat, closed off, navigable, cluttered). Both category systems were developed and validated in prior work, using a different sample of participants (Figure 2C).^17^ To determine whether participants were aware of the disparity manipulation, we also asked them to discriminate the stereoscopic viewing condition (mono, stereo, or reverse-stereo, Figure 2B).

Part 2 of this paper uses a computational analysis of the data from Part 1 to test the second core prediction of space-centered theory, i.e., that a representation of spatial structure is computed as an intermediate step toward semantic categorization.

## Results

**Figure 3:**
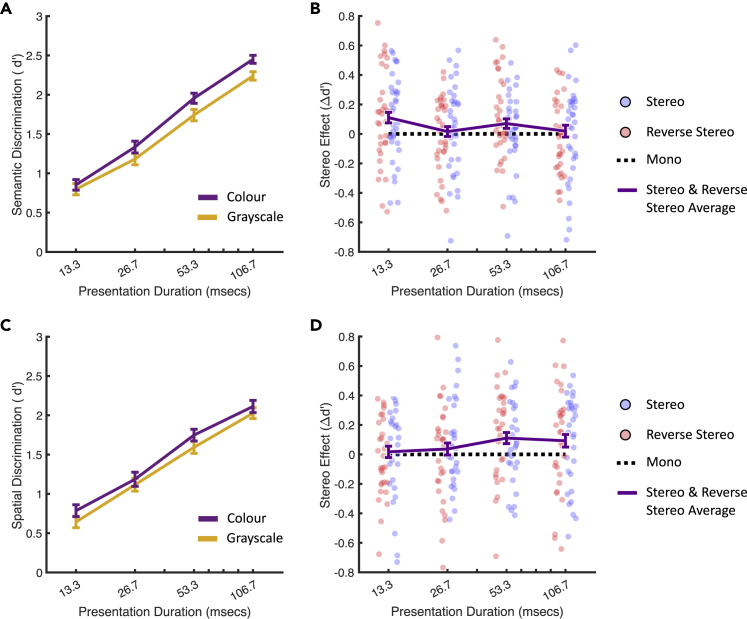
Semantic (A & B) and Spatial Structure (C & D) category discrimination performance, quantified as *d’* (A) Semantic performance as a function of presentation duration and color (collapsed across monocular/binocular viewing conditions). (B) The semantic task stereo effect as a function of presentation time. The stereo effect ($\Delta d^{\prime }$) is defined as the difference between the mono condition and the two stereo conditions. Individual data points represent condition means for individual participants. The purple line shows the average of the stereo and reverse-stereo conditions. Positive values indicate a stereo advantage. (C) Spatial structure performance as a function of presentation duration and color. (D) The spatial structure task stereo effect as a function of presentation duration. All error bars are ±1 standard error (SE) over observers.

In the semantic task, categorization improved as a function of presentation time (Z = 25.16, p <0.001), with above-chance discrimination for all presentation durations, including just 13.3 ms (Z = 9.54, p <0.001). Color improved categorization (Z = 10.33, p <0.001), and color interacted with presentation time (Z = 3.01, p = 0.002), such that longer presentations generated a stronger color advantage. Disparity cues also conferred a small advantage (Z = 2.78, p = 0.005), and this was shared across the stereo and stereo-reversed disparity conditions (full model results in Data S1). The stereo advantage was slightly greater for shorter presentation durations (Z = −1.99, p = 0.046). Figures 3A and 3B show these effects using *d’* – a psychophysical metric of stimulus discriminability, where *d’* = 0 equates to chance. Quantifying performance in terms of *d’* allows a fair comparison between semantic and spatial structure performance because it is insensitive to the number of categories (see Data S1 for computational details).

Categorization of spatial structure also improved as a function of presentation duration (Z = 16.89, p <0.001), with above-chance discrimination for presentation durations from 13.3 ms (Z = 8.61, p <0.001). Color cues improved categorization (Z = 5.13, p <0.001), as did disparity cues (Z = 2.26, p = 0.024, see Data S1). These effects are shown in Figures 3C and 3D.

**Figure 4:**
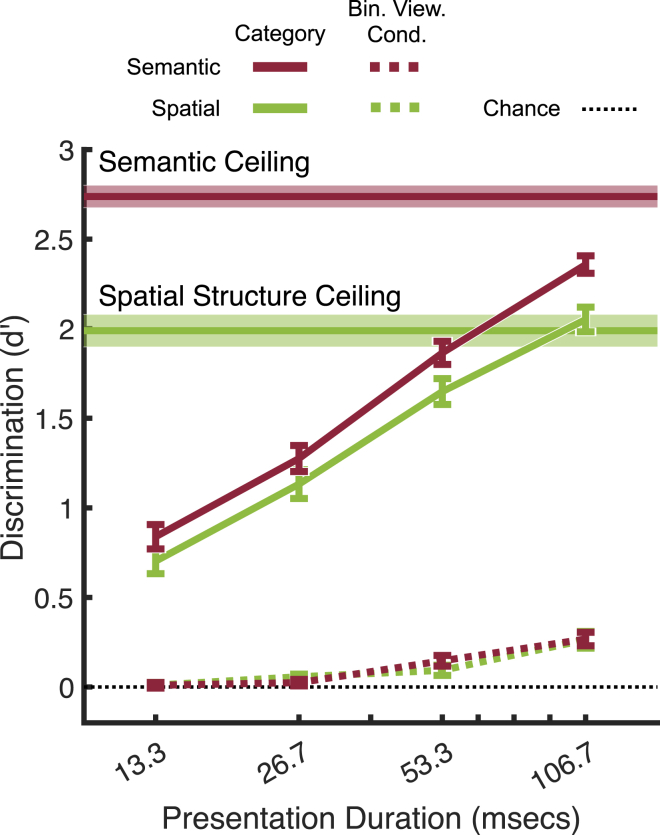
Semantic, spatial structure, and binocular viewing condition discrimination as a function of presentation time, collapsed across color and stereo condition All error bars are ±1 SE over observers. Ceiling performance was derived from a prior study^17^ in which a separate set of participants (*N* = 20 for both category systems) were given unlimited time to view and categorize the images we use here. For each task and every image, we used leave-one-out cross-validation to define ground-truth category labels, using the mode response from 19 (*N*-1) participants and then determined the performance ($d^{\prime }$ values) of the left-out participant by comparing their responses to this ground-truth. This was repeated 20 times, leaving out each participant in turn.

Discrimination of the binocular viewing condition (mono, stereo, stereo-reversed) improved with presentation time (Z = 15.17, p <0.001), but only exceeded chance for durations of 53.3 ms or longer (Z = 7.02, p <0.001). Observers’ discrimination of the binocular viewing condition was substantially worse than semantic and spatial structure discrimination (Figure 4). Note also that absolute performance in semantic discrimination exceeds that for spatial structure discrimination at every presentation duration. However, this requires careful interpretation (see below) because ceiling performance is different in the two tasks (horizontal lines and shaded error bars).

The space-centered approach to real-world scene categorization holds that humans exploit discriminative spatial structure information to predict a scene’s semantic category during early visual processing.^5^^,^^7^ This suggests that the increase in sensitivity to semantic category with increasing stimulus duration should roughly track the increase in sensitivity to spatial category. To test this prediction, we investigated the time-courses of semantic and spatial structure categorization. An unbiased comparison across the two tasks requires a normalized performance scale that adjusts for differences in ceiling performance (see Figure 4).

Ceiling discrimination in both tasks was quantified using data from an independent set of observers and a separate task in which subjects had unlimited time to view and categorize the images (see Figure 4 legend for more details). The resultant estimates of ceiling performance are shown in Figure 4 (horizontal lines); ceiling performance is higher in the semantic task than in the spatial structure task. In other words, when observers are given unlimited time to form a decision, there is greater agreement between participants with respect to the ‘correct’ category for the semantic task than for the spatial structure task. Thus, although semantic discrimination is more accurate than spatial structure discrimination in absolute terms, ceiling performance is reached only in the spatial structure task, and not in the semantic task. This suggests that asymptotic/ceiling discrimination is critical for interpreting differences in task performance.

**Figure 5:**
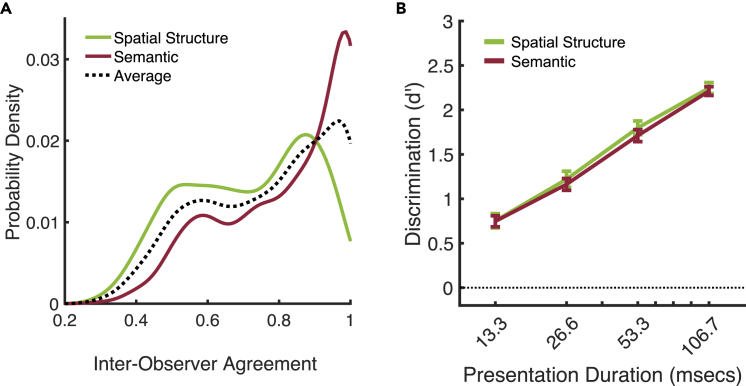
A fair comparison between spatial structure and semantic categorization performance, controlling for differences in inter-observer agreement in the ground-truth dataset (see STAR Methods) (A) Probability distributions of inter-observer agreement in semantic and spatial structure task. The negative skew and greater average agreement in the semantic task precludes a straightforward comparison of absolute $d^{\prime }$ performance. For visualization, histograms were smoothed with a Gaussian kernel (bandwidth = 0.04). (B) Normalized performance (with the same weights across both tasks at every agreement bin – see black dotted line in A reveals that the time-courses for spatial structure and semantic processing are very similar. All error bars are ±1 SE over observers.

The level of inter-observer agreement also varies across individual images: the histogram in Figure 5A shows the distribution, across all images, of the level of inter-observer agreement in categorization, given unlimited viewing time. For any given image, inter-observer agreement is determined as the proportion of ‘votes’ for the mode category. With 20 participants,^17^ chance-level agreement = 1/20, and maximum agreement (i.e., all participants select the same category) = 1. Despite a *larger number of categories*, semantic categorization (Figure 5A, red) produced a greater number of images with very high agreement than spatial structure categorization (green). Images with greater time-unlimited agreement are categorized more accurately in both the semantic (Z = 9.20, p <0.001) and spatial structure time-limited tasks (Z = 11.78, p <0.001, see Data S1).

To control for these differences in inter-observer agreement (i.e., ceiling performance), we first computed $d^{\prime }$ separately for each task and each level of inter-observer agreement. We then computed a weighted average within each task, where the weights were given by the normalized histogram bin averages across the two categorization tasks (Figure 5A). This serves to equalize inter-observer agreement across the semantic and spatial structure tasks. The result is shown in Figure 5B. Whereas *un*normalized performance suggests that semantic discrimination is consistently better than spatial structure discrimination, normalized performance reveals that, when tasks are matched for their ceiling performance, discrimination of semantic and spatial structure unfolds over time in a very similar way (for a break-down of task differences in $d^{\prime }$ per agreement bin, see Figure S6). Using bootstrapping to equalize the number of images/trials in each agreement bin produces the same result (see Figure S7). Moreover, these results are not dependent on the $d^{\prime }$ performance metric; highly similar results are observed when performance is measured using other metrics, including proportion of variance explained by multinomial regression (*R*^*2*^) and mutual information (see Figures S4 and S5). Thus, the increase in sensitivity to semantic category over time tracks the increase in sensitivity to spatial structure, consistent with the space-centered theory of scene categorization.

However, this correlation of performance over time does not prove causation. Certainly, it is consistent with the space-centered theory (spatial structure → semantic) that semantic categories are derived from spatial structure.^1^^,^^2^^,^^16^^,^^27^ But it is also consistent with an ‘independent’ theory of two parallel processes, one for spatial structure and one for semantics, that happen to progress at a similar pace, and with a semantic → spatial structure theory in which spatial categories are derived from semantics.

To distinguish these three possibilities, we performed a finer-grained analysis, examining, for each image, to what degree the perceived spatial category predicts the assigned semantic category, and vice-versa, assuming that the observer has access to a stored model of conditional probabilities. Specifically, we first calculated probabilities of each semantic category conditioned on each spatial category and vice-versa, based on our prior study in which observers categorized images without time constraints,^17^ and used these probabilities to model the stored internal prior knowledge relating spatial and semantic categories. Now, to simulate the spatial structure → semantic model, for each image and stimulus duration in our time-constrained experiment, we predict the perceived semantic category by selecting the mode of the stored prior conditional distribution over semantic categories, conditioned on the spatial category selected for that image and stimulus duration. Similarly, we simulate the semantic → spatial structure model by selecting the mode of the stored prior conditional distribution over spatial categories, conditioned on the semantic category selected for each image and stimulus duration. The two opposing models can then be evaluated by comparing these predictions with the actual semantic and spatial categories assigned for each image and stimulus duration.

Mathematically, for the spatial structure → semantic model, we let $x_{ijk}$ represent the spatial structure category selected for the ith image at the jth presentation time by the kth of *n* observers and let C represent the set of all semantic categories. Let p(y|x) represent the empirical conditional probability of semantic category *y*, given spatial category *x*, derived from the spatial and semantic category labels provided by 20 observers in our time-unlimited task.^17^ We then define the semantic category ${\hat{y}}_{ij}$ predicted for the ith image at the jth presentation time as

(Equation 1) $${\hat{y}}_{ij}=\underset{y\in C}{\arg\;\max\;}\sum_{k=1}^{n}p\left(y|x=x_{ijk}\right)$$

Note that although the empirical conditional probability distribution p(y|x) is based on a pool of observers and unlimited viewing time, the predicted semantic category ${\hat{y}}_{ij}$ is based on the spatial category labels $x_{ijk}$ identified by each observer *k* for limited presentation time *j*, and thus will reflect spatial category biases particular to each observer and time constraint. The semantic → spatial structure model predictions are calculated in the same way, with *y* representing spatial category and *x* representing semantic category.

**Figure 6:**
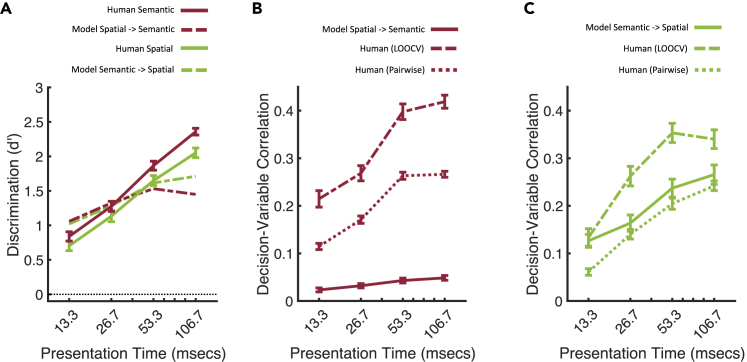
Causal models and human categorization (A) Model and human category discrimination ($d^{\prime }$) as a function of presentation time. (B) Spatial structure → semantic model decision-variable correlation (DVC).^28^ For reference, DVCs between (i) individual humans and the mode responses from N-1 humans (leave-one-out cross-validation; dashed lines), and (ii) pairs of humans (pairwise; dotted lines) are also shown. (C) Semantic → spatial structure model DVC. Note that the semantic → spatial structure model (solid line) is a better predictor of human categorization in terms of absolute DVC, and relative to the two performance references (LOOCV and pairwise). All error bars are ±1 SE over observers.

Figure 6A compares the performance of these two models relative to human performance on the two categorization tasks. Performance of both the semantic → spatial structure and spatial structure → semantic models improve over time (dashed lines). This can be attributed to improvements in categorization within the predictor dimension which, in turn, improves classification performance in the target dimension. The semantic → spatial structure classifier (dashed green line) is slightly more accurate than the spatial structure → semantic classifier (dashed red line) at longer presentations, but both models achieve above-chance discrimination, like humans, from the shortest presentation time.

How can we test whether either of these models is a good predictor of human behavior? It is insufficient to compare $d^{\prime }$ values, because the model and human could produce similar overall discrimination performance, while differing on the specific images that are correctly categorized. As a stronger test, we use decision-variable correlation (DVC),^28^ which is an extension of the standard signal detection theory framework, complementing $d^{\prime }$ and decision criteria/bias. The DVC quantifies the trial-by-trial correlation between human and model responses, while controlling for chance-variation in model-human correlations associated with overall performance. To extract the DVC from a 4AFC and 6AFC task, we begin by converting categorical responses to binary incorrect/correct responses. We then compute the Pearson correlation (also known as the phi coefficient) between model and human incorrect/correct responses, independently for each ground-truth category, and then average over categories. Because the DVC is an average of correlation coefficients, it is bounded between −1 and 1, with a value of zero indicating only chance agreement between human and model (In Data S1, we evaluate an alternative method for adapting DVC to our multinomial tasks, and also assess Cohen’s kappa^29^ as an alternative measure of human-model agreement. All methods yield consistent results – see Figures S8 and S11)

In the semantic categorization task, the DVC between model and observer performance is weak (Figure 6B, solid line). Moreover, we can compare the strength of this model-human relationship to its theoretical ceiling: the extent to which humans predict each other, the human-human DVC. This ceiling can be quantified in two different ways: (1) The DVC between pairs of observers’ raw responses (dotted line); (2) the DVC between a single observer’s responses, and the mode responses from N-1 observers (i.e., leave-one-out cross-validation/LOOCV, dashed line). Figure 6B reveals that the spatial structure →semantic model provides a poor account of human responses in the semantic classification task, suggesting that observers do not use spatial structure to infer the semantic category.

In contrast, Figure 6C shows that the semantic → spatial structure model provides a reasonable account of human behavior (solid green line), and is in fact a better predictor than one human is of another (pairwise human-human DVC, dotted green). This analysis suggests that observers use semantic information to infer spatial layout – i.e., a reversal of the space-centred model.

Note that the superiority of the semantic → spatial structure model, relative to the spatial structure → semantic model cannot be explained by the slightly better $d^{\prime }$ task performance of the semantic → spatial structure model (Figure 6A), because DVC factors out task performance (for an empirical demonstration, see Figure S9).^28^ Nor can this result be explained by differences in inter-observer consistency (reflected in the human x human DVCs; see Figure S10).

Figure 6A shows that for longer presentation times, humans outperform both models, indicating that neither model cannot offer a complete explanation for human spatial categorization for these longer durations. This incompleteness could stem from inter-observer differences in stored prior models, because our prior model was derived from a separate pool of observers. But it could also reflect the later computation of other image properties, used by the brain together with semantic information.

## Discussion

Within a fraction of a second, humans can extract a wealth of information from real-world scenes. In this study, we found that disparity and color information facilitate semantic and spatial structure discrimination shortly after image onset. The disparity advantage was observed for both the stereo and stereo-reversed presentation conditions. Because, in the stereo-reversed condition, figure/ground sign, surface orientation and shape are inverted, this finding suggests that the early stereo advantage is not because of direct 3D shape and/or scene layout information and may instead relate to improved object segmentation. This is consistent with recent work showing that disparity enhances object segmentation in cluttered, naturalistic scenes,^30^ and improves the recognition of objects presented for just 33 ms.^31^

The color advantage observed in our semantic task adds to existing evidence of the contribution of color cues to early scene processing. Different scene categories contain different color distributions (e.g., forests are green/brown, and beaches are yellow/blue), and humans encode these color regularities to inform early semantic scene recognition.^32^^,^^33^^,^^34^ Further work has shown that the advantage of color information may not be limited to global summary statistics (e.g., color histograms): much like disparity, discontinuities in image hue provide a strong segmentation cue.^35^^,^^36^^,^^37^^,^^38^ Our study is the first to demonstrate that color cues provide a similar advantage in spatial structure perception.

The space-centered theory of rapid scene categorization maintains that semantic categories are derived from spatial structure processing. This predicts that sensitivity to spatial structure should emerge earlier, or at least no later, than sensitivity to scene semantics. Although Greene and Oliva^2^ have previously shown that the mean presentation time required to discriminate various spatial structure properties is shorter (34 ms) than semantic categorization (50 ms), our data suggest that humans begin to discriminate both within 13.3 ms. Moreover, once we controlled for steady-state inter-observer agreement, we observed no significant difference between semantic and spatial structure discrimination performance at any presentation time, suggesting that these properties are computed at the *same rate*. Given that we observed substantial task-related differences in discrimination *before* controlling for inter-observer agreement, and thus ceiling performance, our analyses highlight the importance of considering the reliability, across observers, of ground-truth labels when comparing performance across tasks.

Several additional factors may explain why, in contrast to Oliva and Greene,^2^ we found no precedence for spatial discrimination: For example, Oliva and Greene’s^2^ spatial structure task included discrimination between natural and man-made environments. The natural vs manmade distinction is usually classed as a *superordinate semantic* categorization, and superordinate categories are accessed more efficiently than basic-level semantic categories.^39^^,^^40^ Because basic-level categories were used for the semantic task, relative task performance is confounded by differences in decision granularity. Further differences include: (1) the spatial structure properties used, (2) the image database, and (3) the mask properties (in Oliva and Greene,^2^ dynamic masks were created using a texturization algorithm^41^ that preserves the global image statistics of natural images; we used spectral-density-corrected composites of natural images – see Figure S2).

Although the simultaneous emergence of sensitivity to spatial and semantic categories is still consistent with the spatial structure → semantic computation predicted by space-centred theory, it is just as consistent with a reversed semantic → spatial structure computation. Using a more fine-grained analysis, we found that trial-by-trial semantic categorization responses differed dramatically from the predictions of a spatial structure → semantic model. Thus, human semantic categorization is poorly predicted by human spatial structure perception. In contrast, the semantic → spatial structure accurately predicted trial-by-trial human responses, surpassing the correlation between human observers. These results suggest that, instead of using spatial structure properties to predict semantic categories, the human brain employs the opposite strategy, using semantic properties to inform spatial structure discrimination.

This finding contradicts previous evidence of a correlation between human and spatial structure → semantic model categorization.^1^ However, this prior study used a coarser analysis and did not assess the ability of the spatial structure → semantic model to predict human responses for individual images.

The role of semantics, or ‘high-level’ knowledge in perception has been debated for centuries and that debate continues today. The 20^th^ and early 21^st^ centuries, through Gibson^42^ and Marr,^43^ connectionism^44^ and modern deep learning,^45^^,^^46^ have been dominated by the ‘bottom-up’ view that semantic understanding is largely an outcome of a feed forward constructive computation that proceeds systematically from local 2D features to global 3D structures and meaning. The space-centered spatial structure → semantic theory of scene categorization^6^^,^^8^^,^^27^ belongs to this tradition.

However, serious limitations have been found in even the most powerful of these feed forward models,^47^^,^^48^^,^^49^ and there is increasing evidence that the brain relies profoundly on recurrent computations to overcome these limitations.^50^^,^^51^ These recurrent neural circuits, known to play an important role in scene-selective areas of visual cortex,^52^^,^^53^^,^^54^ are a plausible computational substrate for the inference of spatial structure from semantic category processing.

### Limitations of the study

The relative time-course and measured relationship between semantic and spatial structure perception may vary with the choice of category system and image dataset. Our category systems were derived from the SYNS dataset,^55^ which is, at the time of writing, one of the most diverse stereo datasets available. However, though SYNS was carefully designed to fairly sample environmental diversity over land use categories, all images were sampled from southern England, and therefore exclude some scene types observed in other databases such as SUN^56^ or ADE20K,^57^ e.g., deserts, mountains, and canyons. Clearly, the categories employed in the current study do not represent a complete set of semantic or spatial descriptors. Moreover, it is possible that with a different set of categories relative task performance (including our measure of ceiling performance) may differ from the results presented in this study.

Larger datasets such as SUN primarily rely on crowd-sourced photography, which could generate a bias toward highly curated compositions (where a subject of interest is typically centered in the image), that contain meaningful or aesthetically interesting subjects. Greene and Oliva’s^2^ study – one of the main studies that support space-centered theory – employed the SUN dataset, and although there are several other differences between these studies that complicate comparisons (e.g., masks and psychophysical method) it is possible that disagreement between their results and ours regarding the precedence of semantic versus spatial processing derives in whole or part from the differences in image stimuli (and by extension, semantic/spatial structure labels). Replication of our study on the SUN dataset, or other large-scale datasets, is thus a good opportunity for future work.

## STAR★Methods

### Key resources table

**Table:** 

REAGENT or RESOURCE	SOURCE	IDENTIFIER
**Deposited data**		

Original human experimental data and analysis code (R and MATLAB)	Mendeley	https://data.mendeley.com/datasets/mdk86nb42n/2

**Software and algorithms**		

MATLAB	www.Mathworks.com	Release 2018a
R	https://www.R-project.org	Version 4.0.5

### Resource availability

#### Lead contact

Further information and requests for resources will be fulfilled by the lead contact, Matthew Anderson (matt.anderson@soton.ac.uk).

#### Materials availability

This study did not generate new unique reagents or other materials.

### Experimental model and subject details

#### Participants

Seventy-five undergraduate and postgraduate students (13 Male, age range: 18-29) from the University of Southampton participated as volunteers, or in return for course credits. Stereoscopic vision was tested using the Titmus stereo test (Stereo Optical, USA), and all participants were required to have a stereoacuity of at least 40 arcseconds. Ten participants failed this requirement and were excluded. One additional participant in the semantic categorization task was excluded because their performance was worse than three standard deviations below the median. This left thirty-five participants in the semantic categorization task, and 30 in the spatial structure task (65 overall). Informed consent was obtained prior to experimentation, and ethical approval was acquired from the Research Governance Office, University of Southampton.

### Method details

#### Materials

We sampled 708 high-resolution outdoor stereo pairs from the Southampton-York Natural Scenes (SYNS) database.^55^ SYNS samples from 92 natural and man-made scenes, comprising 19 distinct land use types in southern England (e.g., farms, retail parks, coniferous woodland, orchards, wetlands, etc.^55^). SYNS is more diverse than other stereo databases, which typically contain contrived arrangements of ‘still-life’ objects,^59^^,^^60^ or a small number of locations, such as University campuses^59^^,^^61^ or roads,^62^ and is therefore more suitable for investigating natural scene perception in humans.

Ground-truth category labels were derived for all 708 images in an independent study, where a separate group of observers were instructed to sort images based on semantics or spatial structure.^17^ In the semantic task, observers were asked to sort images based on the ‘type of place’, using descriptions they would naturally/intuitively use to describe the scenes (e.g., mountain, desert). In the spatial structure task, observers sorted images by ‘three-dimensional layout’, and were encouraged to consider how, if one were to physically build a 3D model of each scene, the coarse layout of that model may differentiate the stimuli. In both cases, observers assigned free-text labels to every category they created. Observers viewed the stimuli for an unlimited duration. Spatial structure and semantic categories were estimated using the CIRCA clustering algorithm,^17^ which optimizes the agreement (measured via the Rand index) between an estimated group-level category system (over pooled responses from all observers), and the raw categorical judgements of individual observers. Category labels were then identified using the text labels generated by observers (labels were selected based on the agreement with the group-level categories, and frequency of use^17^). The resulting semantic category labels were beach, residential, road, farm, car-park, and nature. The spatial structure category labels were open, closed off, navigable, and cluttered (see Figure 2C). The distribution of SYNS images across both category systems is reported in Figure S1.

In the current experiment, stimuli were presented on a dual-monitor display (two 32-inch, 2560 x 1440 pixel, 75-Hz, ASUS PB328Q monitors) via a single-bounce Wheatstone mirror stereoscope at an effective viewing distance of 83.5 cm. Stimuli subtended 31.12 x 22.36 degrees of visual angle – a scale that matched real-world viewing conditions.^55^ Stimuli were presented for limited durations, with backward masking. Unique masks were generated for every trial by randomly selecting two stereopairs from each scene category (ensuring the target image was *not* sampled), vertically flipping one image in each pair, and creating a composite image (i.e., pixel-wise averaged) containing all 8-12 images. Because these composite images have a lower amplitude at all spatial frequencies than the original images (see Figure S2 for examples), we adjusted the mask’s amplitude spectrum to match the target image. Every category was equally represented in the mask in both tasks; it was thus not possible for participants to use mask properties to estimate the category of the target image. (Note that, even if one visual category dominated the mask percept, trial and mask randomization would eliminate any relationship with the ground-truth category). Colour and grayscale masks were created for the colour and grayscale image conditions, respectively. The experiment was programmed in MATLAB (MathWorks, Inc., Natick, MA).

#### Design & procedure

We employed a 2 (colour: colour/grayscale) X 4 (presentation time: 13.3, 26.7, 53.3, 106.7 msecs) X 3 (binocular viewing condition: mono/stereo/reverse-stereo) within-subjects design for both spatial structure and semantic tasks. Our sample of 708 images were pseudo-randomly assigned to a presentation time and viewing condition, ensuring an equal number of stimuli per condition. Each image was presented twice: once in colour and once in grayscale (see below). Pseudo-random assignment was performed independently for the two colour conditions. There was a total of 1,416 trials. For our binocular viewing condition manipulation, we presented images monoscopically (the left and right eyes viewed the same image), stereoscopically (with correct, real-world disparities), or reverse-stereoscopically (where the left and right eye’s images are swapped; these are also referred to as pseudoscopic images). Reverse-stereo images contain the same relative disparities at object boundaries as stereo images, but the figure-ground depth order is inverted (see Figure 2B). In other words, objects that ‘pop out’ of their background in normal stereoscopic images, now ‘sink’ into their backgrounds like holes. This manipulation preserves the locations of disparity-defined depth discontinuities that might aid segmentation, but reverses the depths. The spatial structure and semantic task procedures differed only in the labels used for category discrimination.

In our time-constrained scene classification experiment, observers began by completing a short, supervised training session where they were familiarized with the task of discriminating mono, stereo, and reverse-stereo images, using longer presentations and a subset of images not included in the experiment. Next, participants completed a short practice session of the main categorization task before beginning the experimental trials.

On each experimental trial, observers were first shown a 1-second fixation screen comprised of a central dot inside a random dot frame, included to assist fusion, both presented at an absolute disparity equal to the disparity of the centre of the target image (Figure 2A). This ensured that the region of the target image near fixation could be fused without any corrective vergence eye movements. For monoscopic stimuli, we adjusted the disparity-defined depth of the entire image to match the depth of the fixation point in the stereoscopic conditions. Thus, the disparity-defined depth of the fixation point and centre of the target image was constant across mono, stereo and reverse-stereo presentation conditions. Fixation disparities did not predict scene category (see Data S1).

The target image was presented for 13.3, 26.7, 53.3, or 106.7 msecs, followed by a mask presented for 150 msecs. Participants subsequently reported the semantic or spatial category and binocular viewing condition, identified to the participants as ‘2D’, ‘3D’, or ‘3D-Reversed’. Responses were made by clicking corresponding labels on the display using a mouse. Participants had unlimited time to respond.

Participants completed the experiment in two sessions (708 trials per session) on separate days. The colour/grayscale condition was manipulated in a blocked design (12 blocks of 59 trials), and block order was randomized between participants. Image order was pseudo-randomized between participants; repetitions of the same image were separated by a minimum of two blocks to minimize priming effects. Each experimental session lasted ∼90 mins.

### Quantification and statistical analysis

#### Statistical analyses

We modelled response accuracy using generalized linear mixed models (GLMMs), in R (R Core Team, 2018), with the lme4 package.^63^ Category discrimination and binocular viewing condition discrimination were modelled independently, but in both cases, we used a log-odds link function on the binomial response data (single-trial responses were either correct or incorrect). To account for individual participant and stimulus effects, we specified participant and stimulus (i.e., image) as random effects in all our models (images were nested within categories). Model likelihood was estimated using Laplace approximation.^64^ We maximised the number of terms in the random structure while ensuring model convergence.^65^^,^^66^ For the category discrimination data, we fit random intercepts for participants and stimuli, and correlated slopes for the effect of presentation time as a function of participant (this was the largest random structure that didn’t lead to overfitting, as indicated by failure to converge or singular fit). In other words, we allowed average performance to vary between participants and between stimuli, and we allowed the effect of presentation time to also vary between participants (for the regression equation, see Data S1).

For the binocular viewing condition discrimination, the data only supported a model that included random intercepts for participant and stimulus. Presentation time, colour, and viewing condition were all modelled as fixed effects. Fixed effects are of principal theoretical interest and are reported in the main text. Fixed and random effects are tabulated in full in Data S1. We quantified performance using a generalized form of $d^{\prime }$ that can be computed for tasks with more than two categories (see Data S1).

## Data Availability

Original human experimental data, and R and MATLAB analysis scripts, have been deposited online (Mendeley Data: https://doi.org/10.17632/mdk86nb42n.2) key resources table, and are publicly available as of the date of publication. Any additional information required to reanalyze the data reported in this paper is available from the lead contact upon request.

## Supplemental information

Document S1. Figures S1–S11 and Tables S1–S9

Data S1. Further information on behavioral metrics
