# Supplementary material for: The time-course of real-world scene perception: Spatial and semantic processing
Source: iScience. 2022 Nov 19;25(12):105633. doi: 10.1016/j.isci.2022.105633 (PMC9732406; doi:10.1016/j.isci.2022.105633)
Supplement: Document S1. Figures S1–S11 and Tables S1–S9 [file mmc1.pdf]

iScience, Volume *25*

Supplemental information

The time-course of real-world scene perception: Spatial and semantic processing

Matt D. Anderson, James H. Elder, Erich W. Graf, and Wendy J. Adams

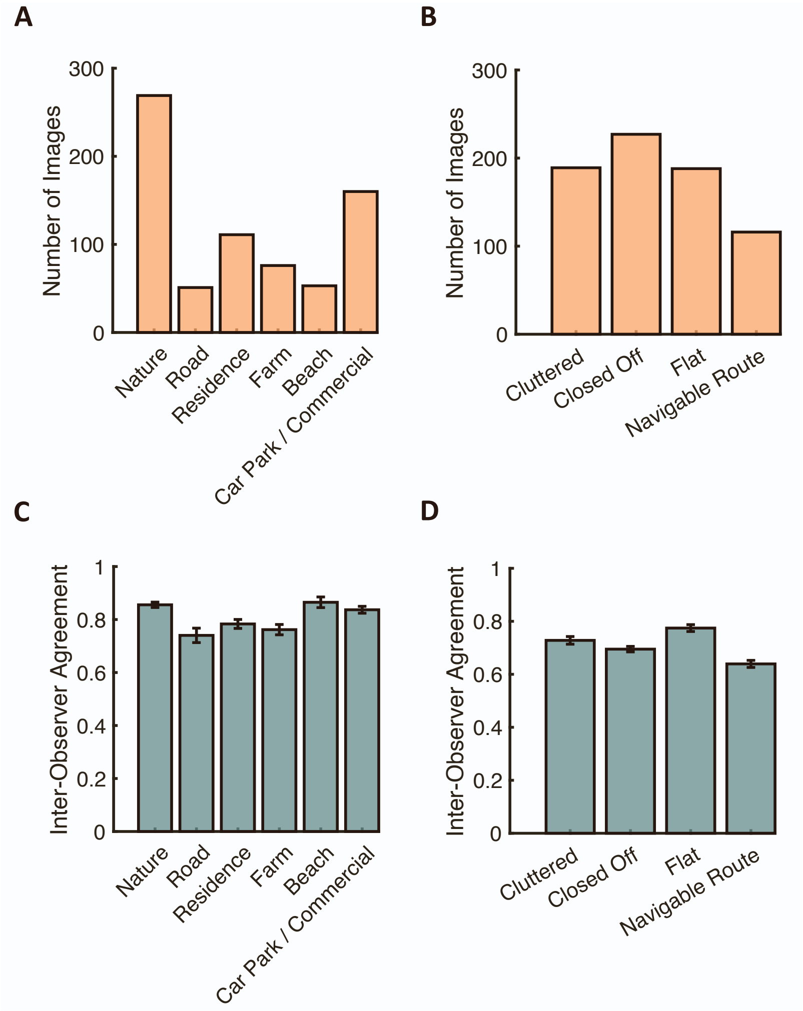

Figure S1. Properties of the semantic and spatial structure category systems, related to Figure 2C. (A) Distribution of images across the semantic categories. (B) Distribution of images across the spatial structure categories. (C) Inter-observer agreement (proportion of observers who selected the mode category) across the semantic categories, for 20 observers who viewed the images for an unlimited presentation duration[1]. (D) Inter-observer agreement across the spatial structure categories. All error bars are ±1 standard error over images.

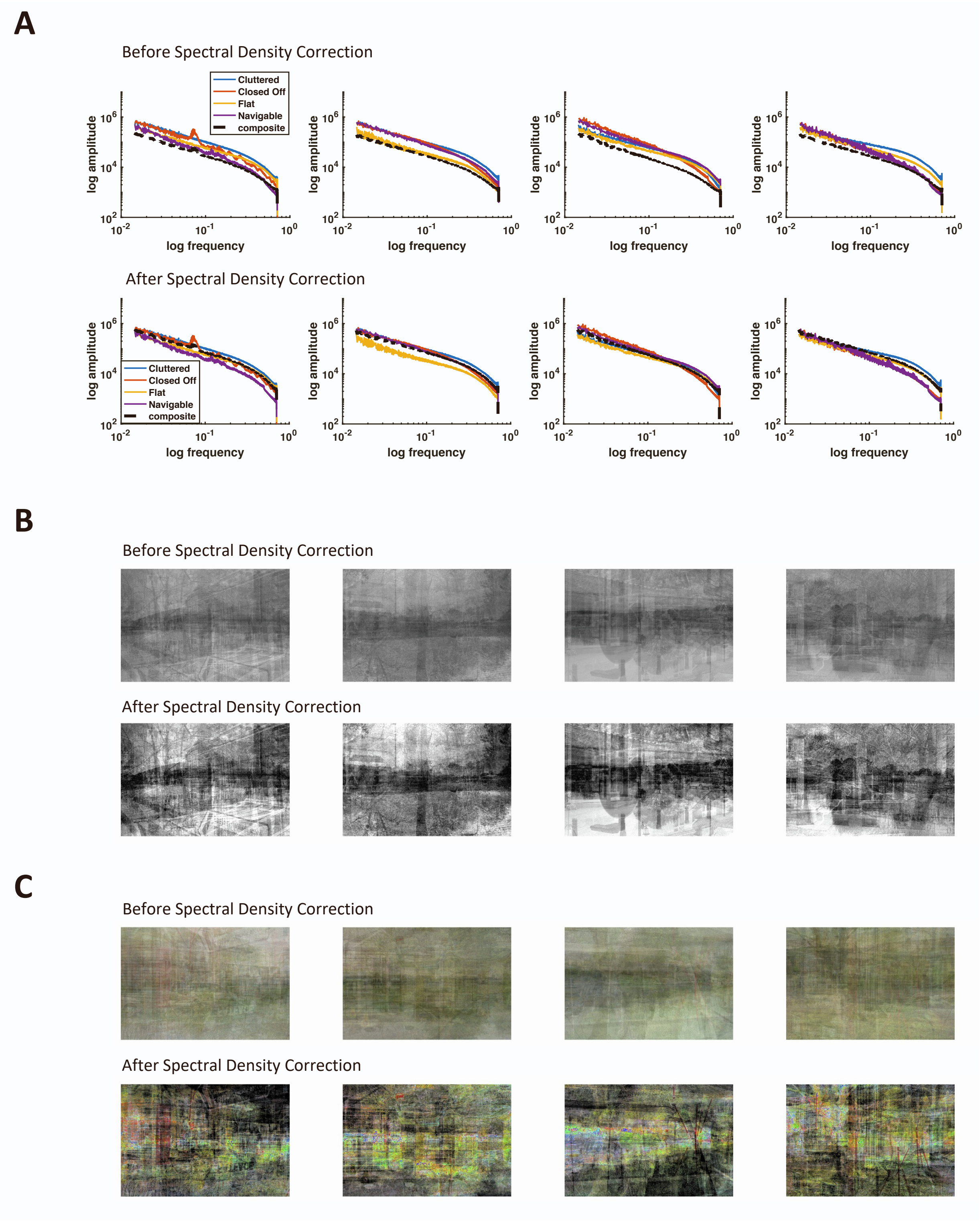

Figure S2. Amplitude spectra of uncorrected masks and corrected masks used in the spatial structure task, related to Figure 2A. Masks were constructed by coercing composite images to have the same amplitude spectrum as the individual images. (A) First row: amplitude spectra of grayscale composite

images versus individual grayscale images, for four composites shown in B, top row. Second row: amplitude spectra of the corrected composite images. (B) Examples of uncorrected (top row) and corrected (bottom row) grayscale masks. (C) Examples of uncorrected (top row) and corrected (bottom row) colour masks.

Table S1. *Semantic Task GLMM Fixed Effect Parameter Estimates, Related to Figure 3.*

Fixed Effect	Logit	Wald 95% CI	SE	Z	Variance Explained (%)
Reverse-Stereo vs Stereo (View 0)	-.004	[-.06 - .05]	.03	-.15	.00
Mono vs Reverse-Stereo & Stereo (View 1)	.07	[.02 - .11]	.02	**2.78****	.01
Grayscale vs Colour (Colour)	.24	[.19 - .28]	.02	**10.33*****	.20
Presentation Duration (PresTime)	1.11	[1.02 - 1.20]	.04	**25.16*****	10.30
View 0 x Colour	-.03	[-.09 - .02]	.03	-1.14	.00
View 1 x Colour	.04	[-.01 - .09]	.02	1.65	.00
View 0 x PresTime	.03	[-.04 - .10]	.04	.77	.00
View 1 x PresTime	-.07	[-.13 - -.001]	.03	**-1.99***	.00
Colour x PresTime	.09	[.03 - .15]	.03	**3.01***	.01
View 0 x Colour x PresTime	-.03	[-.10 - .05]	.04	-.76	.00
View 1 x Colour x PresTime	-.01	[-.07 - .06]	.04	-.16	.00

Note: * p < .05 ** p < .01 *** p < .001

R model equation: $Y \sim PresTime + Colour + View + PresTime{:}Colour + PresTime{:}View + Colour{:}View + PresTime{:}Colour{:}View + (1 + PresTime|Participant) + (1 + PresTime|Stimulus)$.

Table S2. *Post-Hoc Tests of Above-Chance Semantic Category Discrimination per Presentation Duration, Related to Figure 3*

Fixed Effect	Logit	SE	Z	Variance Explained (%)
13.3 msecs	.97	.10	**9.54*** **	3.00
26.7 msecs	1.59	.10	**15.59*** **	8.00
53.3 msecs	2.38	.10	**23.42*** **	18.17
106.7 msecs	3.07	.10	**29.90*** **	29.97

Note: * $p < .05$ ** $p < .01$ *** $p < .001$.

p – values are adjusted using the Bonferroni correction. All fixed effects aside from presentation time were dropped from the model, and the random slopes were removed to enable convergence.

R model equation: $Y \sim PresTime + (1|Participant) + (1|Stimulus)$

Table S3. *Spatial Structure Task GLMM Fixed Effect Parameter Estimates, Related to Figure 3.*

Fixed Effect	Logit	Wald 95% CI	SE	Z	Variance Explained (%)
Reverse-Stereo vs Stereo (View 0)	-.03	[-.02 - .09]	.03	1.23	.00
Mono vs Reverse-Stereo & Stereo (View 1)	.06	[.01 - .10]	.02	**2.26****	.01
Grayscale vs Colour (Colour)	.11	[.07 - .16]	.02	**5.13*****	.06
Presentation Duration (PresTime)	.73	[.65 - .82]	.04	**16.89*****	5.85
View 0 x Colour	.02	[.-.04 - .07]	.03	.61	.00
View 1 x Colour	-.04	[-.09 - .01]	.02	-1.61	.00
View 0 x PresTime	-.03	[-.10 - .05]	.03	-.71	.00
View 1 x PresTime	.01	[.-.05 - .08]	.03	.45	.00
Colour x PresTime	-.02	[-.08 - .04]	.03	-.64	.00
View 0 x Colour x PresTime	-.02	[-.09 - .06]	.04	-.42	.00
View 1 x Colour x PresTime	.02	[-.04 - .09]	.03	.70	.00

Note: * p < .05 ** p < .01 *** p < .001

R model equation: $Y \sim PresTime + Colour + View + PresTime{:}Colour + PresTime{:}View + Colour{:}View + PresTime{:}Colour{:}View + (1 + PresTime|Participant) + (1 + PresTime|Stimulus)$

Table S4. *Post-Hoc Tests of Above-Chance Spatial Structure Category Discrimination at Every Presentation Duration, Related to Figure 3.*

Fixed Effect	Logit	SE	Z	Variance Explained (%)
13.3 msecs	.70	.08	8.61	1.94
26.7 msecs	1.15	.08	14.15	5.24
53.3 msecs	1.70	.08	**20.79**	11.37
106.7 msecs	2.12	.08	**25.80**	17.69

Note: * p < .05 ** p < .01 *** p < .001.

p – values are adjusted using the Bonferroni correction. All fixed effects aside from presentation time were dropped from the model, and the random slopes were removed to enable convergence.

R model equation: $Y \sim PresTime + (1|Participant) + (1|Stimulus)$

Table S5. *Binocular Viewing Condition Task GLMM Fixed Effect Parameter Estimates, Related to Figure 4.*

Fixed Effect	Logit	Wald 95% CI	SE	Z	Variance Explained (%)
Grayscale vs Colour (Colour)	-.0004	[-.03 - .03]	.01	-.02	.00
Presentation Duration (PresTime)	.13	[.11 - .15]	.01	**14.51*** **	3.10
Task	-.01	[-.06 - .05]	.03	-.22	.00
Task x Colour	-.01	[-.04 - .01]	.01	-1.05	.00
Colour x PresTime	.00	[-.03 - .04]	.02	.17	.00
Task x PresTime	-.02	[-.05 - .02]	.02	-.86	.00
Task x Colour x PresTime	.005	[-.03 - .04]	.02	.26	.00

Note: * p < .05 ** p < .01 *** p < .001

R model equation: $Y \sim Task + PresTime + Colour + \ Task{:}PresTime + \ Task{:}Colour + PresTime{:}Colour + Task{:}PresTime{:}Colour + \ (1|Participant) + \ (1|Stimulus)$

Table S6. *Post-Hoc Tests of Above-Chance Binocular Viewing Condition Discrimination at Every Presentation Duration, Related to Figure 4.*

Fixed Effect	logit	SE	Z	Variance Explained (%)
13.3 msecs	.01	.02	.49	.00
26.7 msecs	.04	.02	2.21	.00
53.3 msecs	.13	.02	**7.02***	.10
106.7 msecs	.28	.02	**15.36***	.45

Note: * $p < .05$ ** $p < .01$ *** $p < .001$.

p – values are adjusted using the Bonferroni correction. All fixed effects aside from presentation time were dropped from the model, but the random structure was held constant.

R model equation: $Y \sim PresTime + (1|Participant) + (1|Stimulus)$

Table S7. *Random Effect Estimates for the Semantic Task Category Discrimination Data, Related to Figure 3.*

		SD	Correlation
Random Intercept	Stimulus	1.94	-
	Participant	.27	-
Random Slope	Stimulus x PresTime	.65	.59
	Participant x PresTime	.19	-.26

Table S8. *Random Effect Estimates for the Spatial Structure Task Category Discrimination Data, Related to Figure 3.*

		SD	Correlation
Random Intercept	Stimulus	1.05	-
	Participant	.39	-
Random Slope	Stimulus x PresTime	.56	.51
	Participant x PresTime	.19	-.04

Table S9. *Random Effect Estimates for the Binocular Viewing Condition Discrimination Data, Related to Figure 4.*

		SD
Random Intercept	Stimulus	.10
	Participant	.09

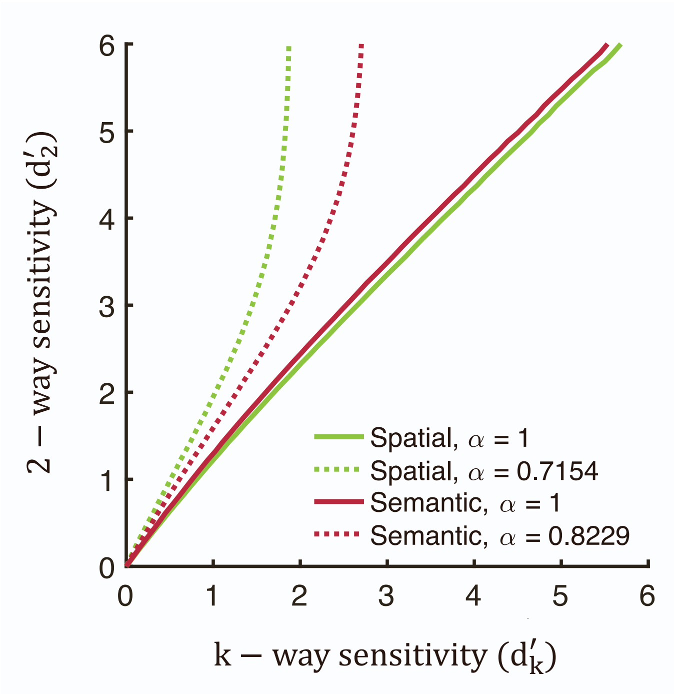

Figure S3. Simulated 2-way sensitivity (d'_2) as a function of d'_k, related to Figures 3 through 6. α is inter-observer agreement on ground-truth category, derived from Anderson et al[1].

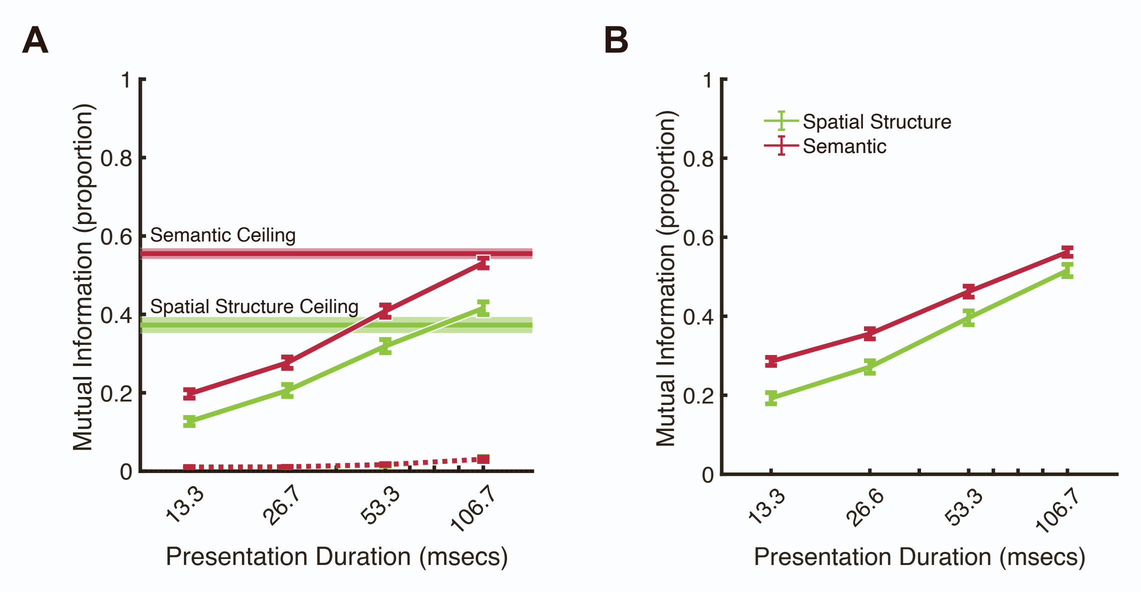

Figure S4. Task performance in the spatial and semantic tasks quantified in terms of mutual information (proportion relative to maximum), related to Figure 5. (A) Spatial structure (green) and semantic (red) category (solid line) and depth (dashed line) discrimination performance. Ceiling performance was determined as for d' (see Fig. 4 in manuscript). (B) Task differences after correcting for differences in inter-observer agreement on the ground-truth category (using histogram equalization – see main text for method). All error bars are ±1 standard error over observers.

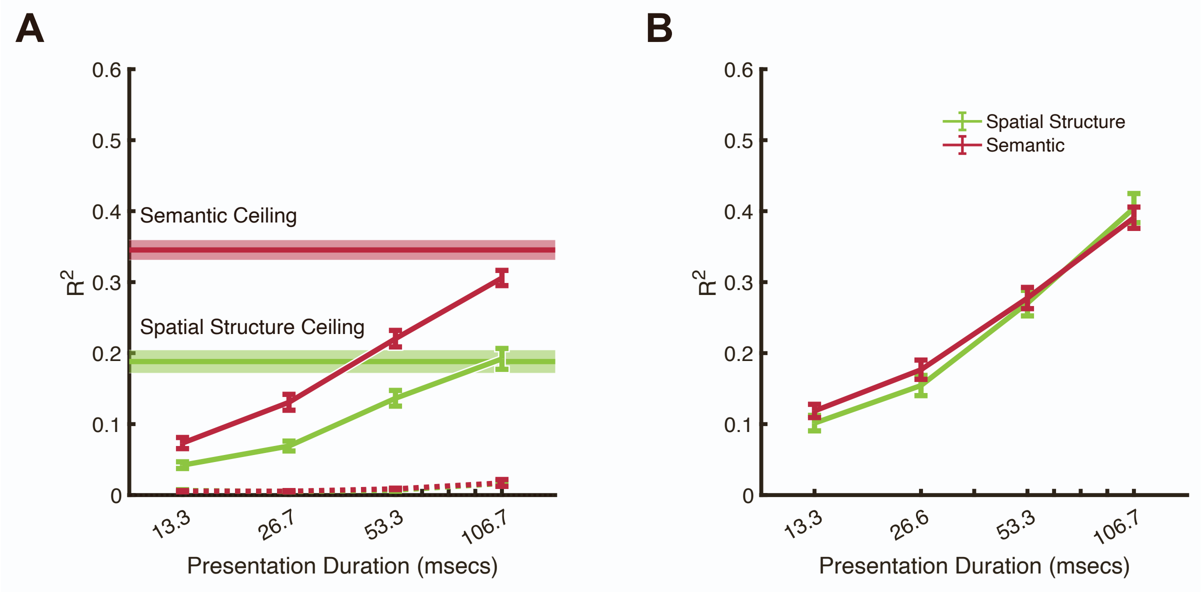

Figure S5. Task performance in the spatial and semantic tasks quantified in terms of variance explained by a multinomial logistic regression (with ground-truth category as predictor), related to Figure 5. (A) Spatial structure (green) and semantic (red) category (solid line) and depth (dashed line) discrimination performance. Ceiling performance was determined as for d' (see Fig. 4 in manuscript). (B) Task differences after correcting for differences in inter-observer agreement regarding the ground-truth category (using histogram equalization – see main text for method). All error bars are ±1 standard error over observers.

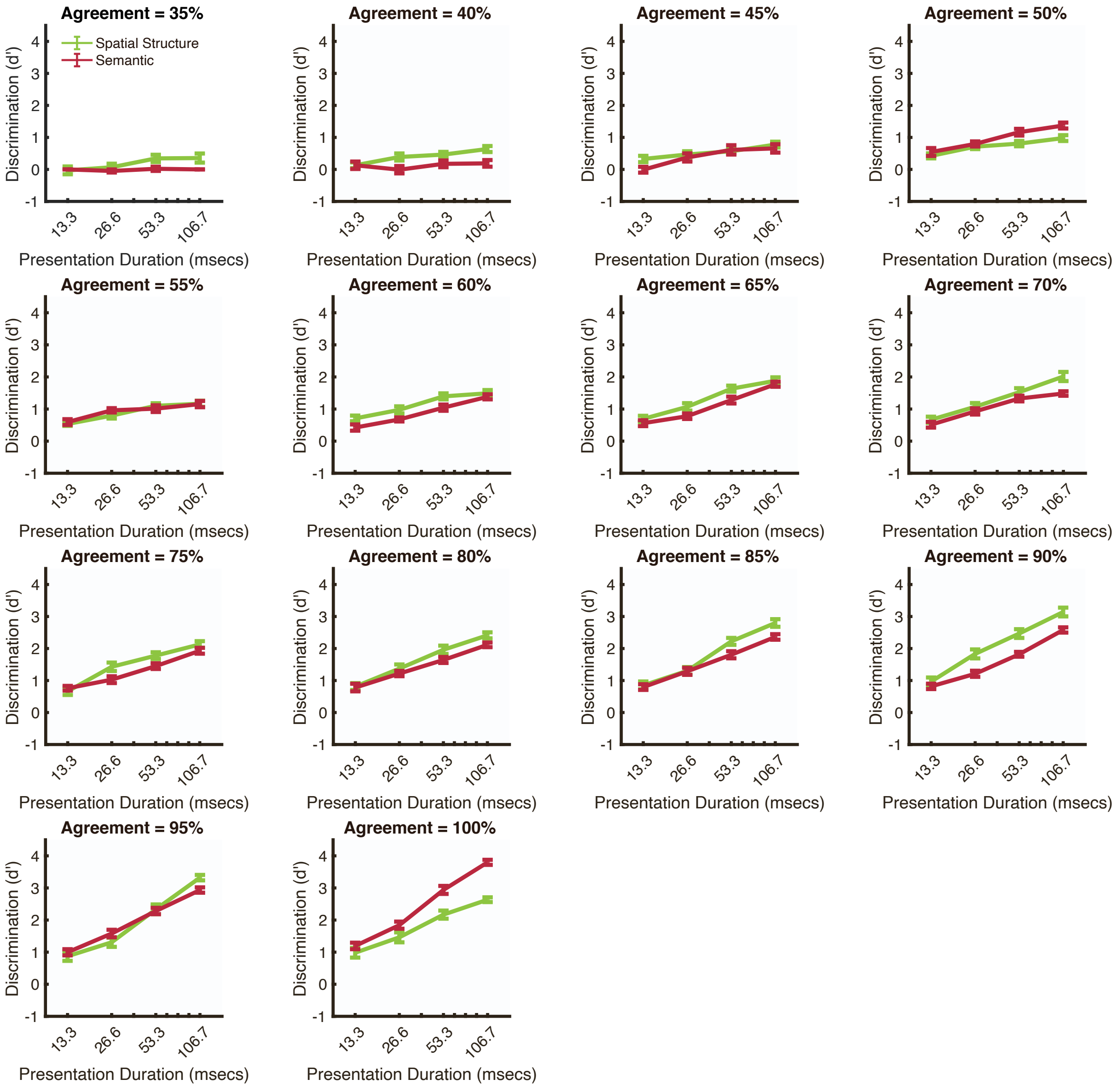

Figure S6. Performance as a function of presentation time, divided by agreement, related to Figure 5. On average, performance is roughly the same on the semantic and spatial structure tasks.

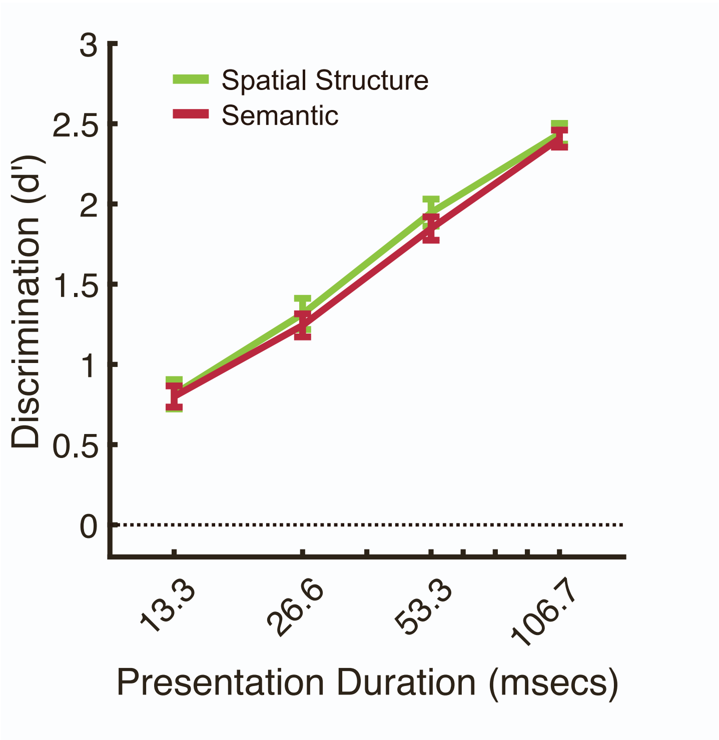

Figure S7. Mean performance after controlling for differences in inter-observer agreement in the ground-truth dataset, related to Figure 5B. Normalized performance (with the same number of trials in semantic and spatial agreement bins) reveals that the time-courses for spatial structure and semantic processing are very similar. All error bars are ±1 standard error over observers.

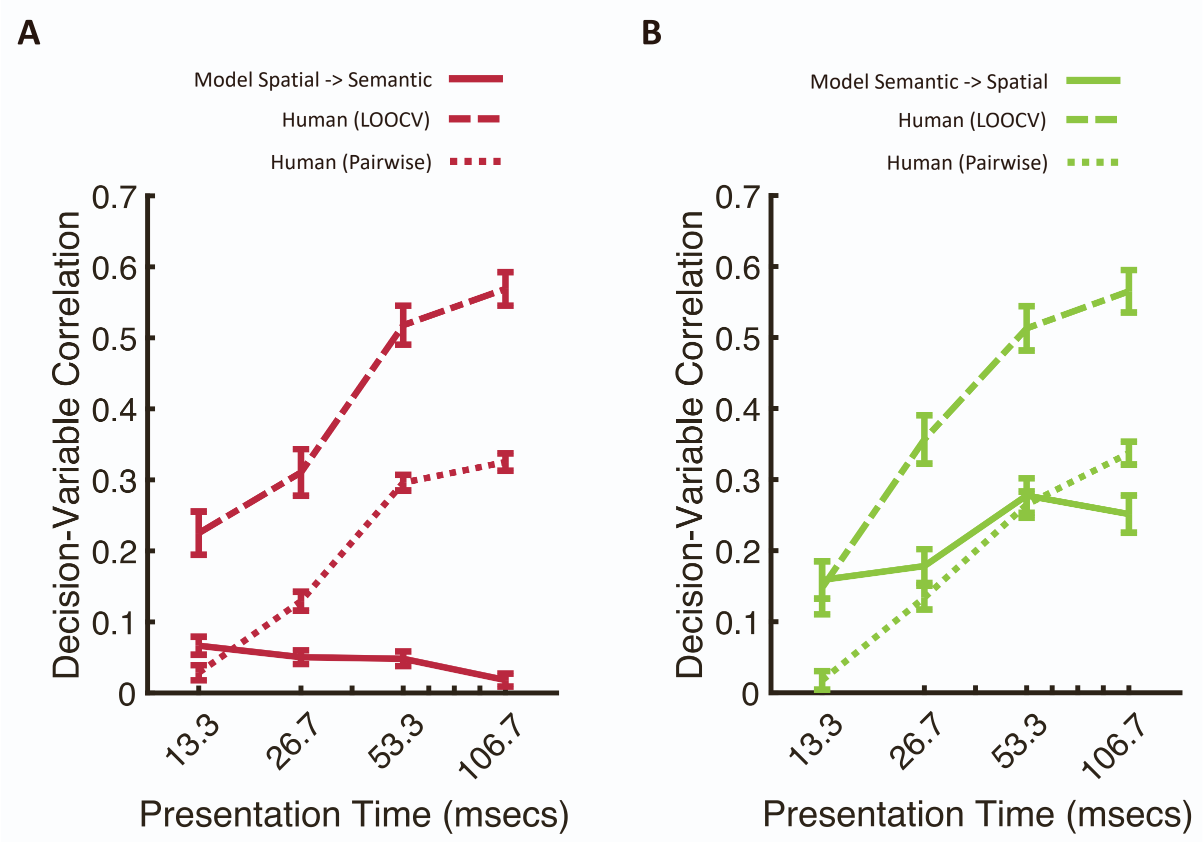

Figure S8. The Decision Variable Correlation (DVC) between human categorization responses and the two models, using a one-vs-all DVC calculation, related to Figure 6. (A) DVC between human semantic responses, and spatial structure → semantic model predictions (solid line). For reference, we also plot the DVCs between (i) individual humans and the mode responses from N-1 humans (leave-one-out cross-validation; dashed lines), and (ii) pairs of humans (pairwise; dotted lines). (B) DVC between human spatial structure responses and semantic → spatial structure model responses. Note that the semantic → spatial structure model (solid line) is a better predictor of human categorization in terms of absolute DVC, and relative to our two performance references (LOOCV and pairwise). Error bars are subject standard errors.

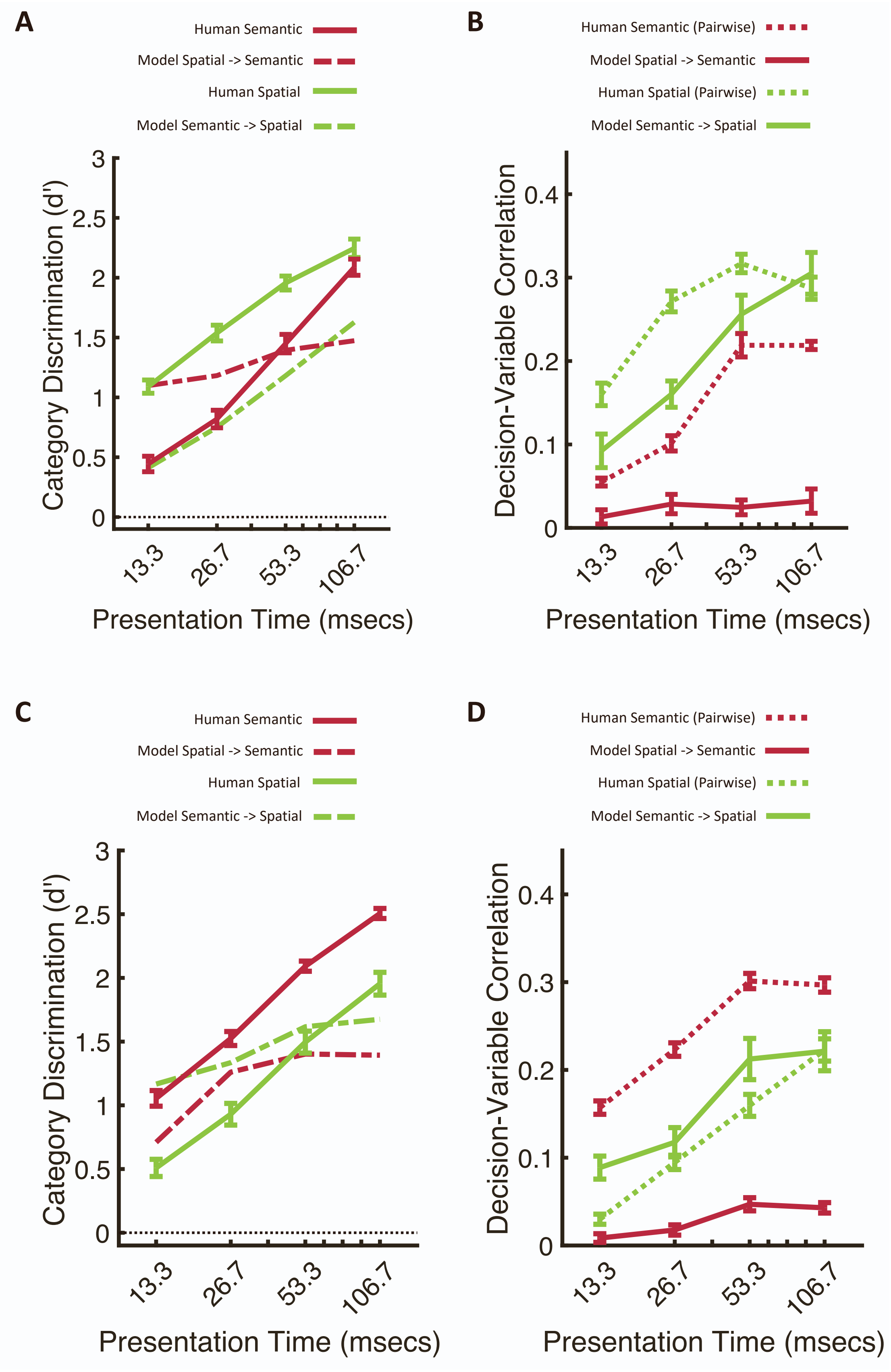

A
Human Semantic
Model Spatial -> Semantic
Human Spatial
Model Semantic -> Spatial
Category Discrimination (d')
0
0.5
1
1.5
2
2.5
3
13.3
26.7
53.3
106.7
Presentation Time (msecs)
B
Human Semantic (Pairwise)
Model Spatial -> Semantic
Human Spatial (Pairwise)
Model Semantic -> Spatial
Decision-Variable Correlation
0
0.1
0.2
0.3
0.4
13.3
26.7
53.3
106.7
Presentation Time (msecs)
C
Human Semantic
Model Spatial -> Semantic
Human Spatial
Model Semantic -> Spatial
Category Discrimination (d')
0
0.5
1
1.5
2
2.5
3
13.3
26.7
53.3
106.7
Presentation Time (msecs)
D
Human Semantic (Pairwise)
Model Spatial -> Semantic
Human Spatial (Pairwise)
Model Semantic -> Spatial
Decision-Variable Correlation
0
0.1
0.2
0.3
0.4
13.3
26.7
53.3
106.7
Presentation Time (msecs)

Figure S9. Model and human categorization patterns when model performance is manipulated, related to Figure 6. (A) Participants are sampled to produce a spatial → semantic model that outperforms the semantic → spatial model. (B) Decision Variable Correlation (DVC) between the two models of categorization (in A), and trial-level categorization judgements. Even though the semantic → spatial model produces worse performance, it still predicts human data better than the structure → semantic model. (C) Participants are sampled to produce a semantic → spatial model that outperforms the spatial → semantic model. (D) DVC between the two models of categorization (in C), and trial-level categorization judgements. The semantic → spatial model predicts human data better than the structure → semantic model. Error bars are subject standard errors.

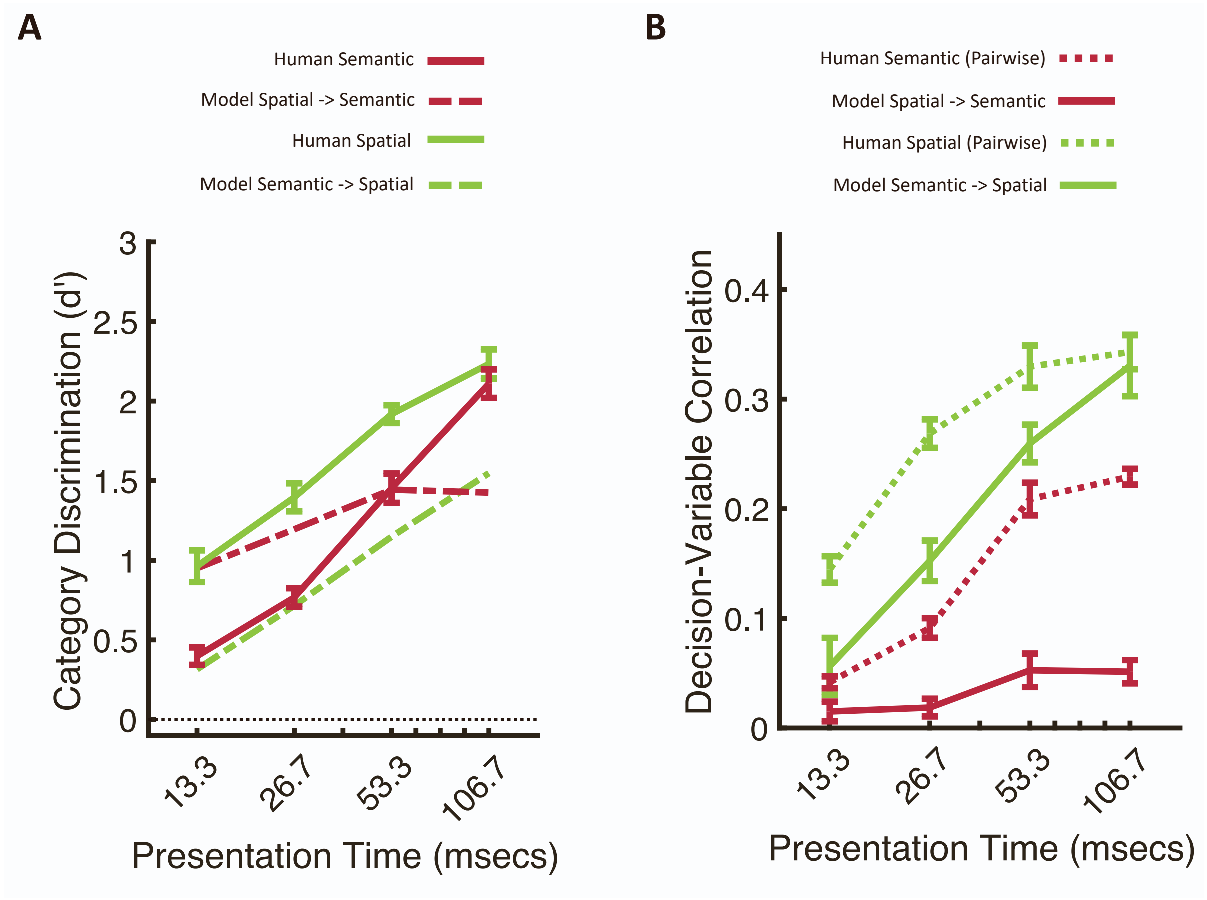

Figure S10. Model and human categorization patterns when human x human DVCs are higher in the spatial task than the semantic task, related to Figure 6. (A) Model and human category discrimination (d') as a function of presentation time. On average, the spatial → semantic model now outperforms the semantic → spatial model. (B) Decision Variable Correlation (DVC) between the two models of categorization, and trial-level categorization judgements. Even though the semantic → spatial model produces worse performance, it still outperforms the spatial → semantic model. All error bars are ±1 standard error over images.

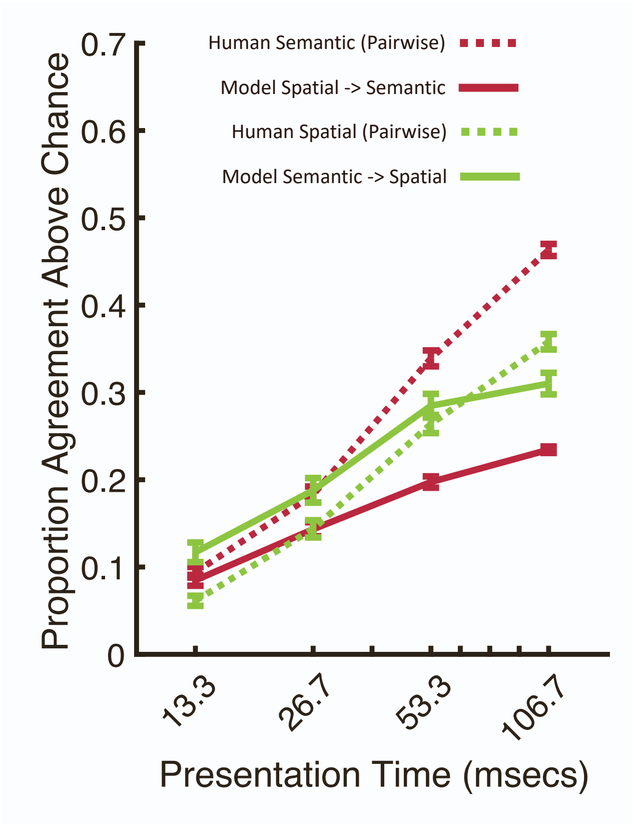

Figure S11. Human-human (dotted) and model-human (solid) agreement quantified as proportion agreement above chance, related to Figure 6A. All error bars are ±1 standard error over images.

Supplemental References

1. Anderson, M.D., Graf, E.W., Elder, J.H., Ehinger, K.A., and Adams, W.J. (2021). Category systems for real-world scenes. Journal of vision *21*, 8-8.
