# Supplementary material for: The time-course of real-world scene perception: Spatial and semantic processing
Source: iScience. 2022 Nov 19;25(12):105633. doi: 10.1016/j.isci.2022.105633 (PMC9732406; doi:10.1016/j.isci.2022.105633)
Supplement: Data S1. Further information on behavioral metrics [file mmc2.pdf]

Mask generation, related to STAR methods

To construct masks, two images were randomly sampled from each category, and one of each pair was rotated by 180 degrees. Images were then pixel-wise averaged to form a composite. The composite image has lower energy at all spatial frequencies, relative to the original images (Fig. S2A), appearing 'washed out' (Figs S2B and S2C, top row). To correct this, the amplitude at each spatial frequency of the composite images was adjusted to match the average amplitude of the individual, original images (see S2A, compare top and bottom rows). For colour images, the RGB channels were adjusted independently. This procedure ensures that the spectral slope of the target image and mask is highly similar, and that the mask contains sufficient image structure (i.e., phase information) - two image properties critical for maximizing mask effectiveness[1].

Generalized linear mixed model, related to STAR methods

In both category discrimination tasks, all experimental manipulations were *within participants.* Participant and image stimulus were crossed random effects. Presentation time, colour, and viewing condition were fixed effects.

Thus the regression model is:

$$(1) \qquad g(\mu) = g\left(E\left[y_{ij}|u\right]\right)$$

$$= \left(\beta_0 + v_{0i} + w_{0j}\right) + \left(\beta_1 + v_{1i} + w_{1j}\right)PresTime_{ij} + \beta_2 Colour_{ij} + \beta_3 View_{ij} + \beta_4\left(PresTime_{ij} \times Colour_{ij}\right) + \beta_5\left(PresTime_{ij} \times View_{ij}\right) + \beta_6\left(Colour_{ij} \times View_{ij}\right) + \beta_7\left(PresTime_{ij} \times Colour_{ij} \times View_{ij}\right) + e_{ij}$$

Here, y_{ij} is the outcome (category decision incorrect or correct, represented as a 0 and 1 respectively) for the ith participant and jth image, and $\mu = E\left[y_{ij}|u\right]$ is its expectation given independent variables u. $g(\mu)$ is the logistic link function $g(\mu) = \ln\frac{\mu}{1-\mu}$. β_0 is the fixed intercept, and v_{0i} and w_{0j} are the random participant and image intercepts, respectively. β_1 is the fixed effect slope and v_{1i} and w_{1j} are the random participant and image slopes for presentation time. $PresTime_{ij}$ is the presentation duration for the ijth trial. β_2 is the fixed effect slope for the colour effect. $Colour_{ij}$ gives the colour condition for the ijth trial. β_3 is the fixed effect slope for viewing condition. $View_{ij}$ gives the viewing condition for the ijth trial (either mono, stereo, or reversed-stereo). Using the $View$ variable, we conducted two a-priori sum contrasts: we

compared the mono condition against the two stereo conditions (ocularity), and we compared the stereo condition against the stereo-reversed condition (disparity sign).

All subsequent terms are interactions between these fixed effects, except e_{ij}, which is the residual error term. In R (lme4), this model can be written as: $Y \sim PresTime + Colour + View + PresTime{:}Colour + PresTime{:}View + Colour{:}View + PresTime{:}Colour{:}View + (1 + PresTime|Participant) + (1 + PresTime|Stimulus)$.

Binocular viewing condition discrimination is modelled differently. Viewing condition is no longer included as a predictor (since viewing condition defines the ground-truth), no random slope terms are included (to enable convergence), and task is a between-subjects variable. This regression model can be defined similarly to Eq. 1:

$$(2) \quad g(\mu) = g(E[y_{ij}|u])$$

$$= (\beta_0 + v_{0i} + w_{0j}) + \beta_1 Task_i + \beta_2 PresTime_{ij} + \beta_3 Colour_{ij} + \beta_4 (Task_i \times PresTime_{ij}) + \beta_5 (Task_i \times Colour_{ij}) + \beta_6 (PresTime_{ij} \times Colour_{ij}) + \beta_7 (Task_i \times PresTime_{ij} \times Colour_{ij}) + e_{ij}$$

where β_1 is the fixed slope for the between-subjects effect of task, and $Task_i$ is the task that the ith participant performed. All other terms are described above. In R (lme4), this model can be written as: $Y \sim Task + PresTime + Colour + Task{:}PresTime + Task{:}Colour + PresTime{:}Colour + Task{:}PresTime{:}Colour + (1|Participant) + (1|Stimulus)$.

Table S7 shows the random effect parameter estimates in the semantic task. Stimuli explain more performance variability than participants. Correlation parameters show the relationship between random intercepts and the effect of presentation time. Images that were, on average, easier to discriminate produced a stronger effect of presentation time ($r = .59$), but participants that performed better, on average, produced a weaker effect of presentation time ($r = -.26$). This is to be expected if the better participants reach ceiling performance before the longest presentation duration.

See Table S8 for the random effect parameter estimates in the spatial structure task. As in the semantic task, stimuli were a greater source of variability in performance than participant. Moreover, images that were, on average, easier to discriminate produced a stronger effect of presentation time ($r = .51$), but there was a weak relationship between participant performance and presentation time ($r = -.04$). This effect might be caused by the difficulty of the spatial structure task, relative to the semantic task (see main text).

Generalization of *d'* to an arbitrary number of categories, related to Figures 3, 4, 5, and 6

For yes/no tasks, in which an observer estimates the category of a presented stimulus, d' is a standard measure of sensitivity that is invariant to response bias. Here we seek a generalization of d' to multinomial classification tasks that retains this invariance to bias, but also allows sensitivity to be compared fairly between tasks with a different number of classes.

Let x represent the ground-truth category, and let y represent the observer judgement for a k-category classification task. We define the per-category hit rate H_c, and false-alarm rate, FA_c, as

(3) $$H_c = p(y = c | x = c)$$

(4) $$FA_c = p(y = c | x \neq c)$$

Sensitivity to category c is then defined as

(5) $$d'_c = F^{-1}(H_c) - F^{-1}(FA_c)$$

where F^{-1} is the inverse of the cumulative distribution function for the normal distribution. Finally, d' for the k-category task is defined as

(6) $$\mathrm{d}'_{\mathrm{k}} = \sum_c p(x = c)\, d'_c$$

Since d'_c is invariant to bias, d'_{k} will also be invariant to bias.

Our aim is to measure the degree to which this definition of d'_{k} enables a fair performance comparison between tasks with different numbers of classes, in our case the semantic (6 categories) and spatial structure (4 categories) categorization tasks. To this end, we conduct a series of simulations. We model category decisions made over a k-category system as emerging from noisy internal k-dimensional response vectors $\tilde{y}$. An image from category i generates a standardized normal vector $\tilde{y}_i \in \mathbb{R}^k \sim \mathcal{N}(\mu_i, I)$, where μ_i is the mean response for category i and the covariance I is the $k \times k$ identity matrix. To model an observer who discriminates any pair of categories with the same pairwise sensitivityd'_2, we specify μ_i to form the vertices of a regular k-dimensional simplex in $\mathbb{R}^k$ with side length of d'_2. We simulate a dataset of 1 million images, drawing categories fairly from the empirical category distribution of our dataset (see Figure S1).

Fig. S3 (solid curves) shows that the relationship between d'_{k} and d'_2 is very similar, but not identical, for spatial and semantic tasks. However, a second complication in comparing observer sensitivity across spatial and semantic categories under time-constraints is variation in the reliability of ground-truth. We derive ground-truth from prior work (Anderson *et al,* 2021)[2] in which observers

categorized images without time constraints. With unlimited time, two observers do not always agree, and we find that the extent of inter-observer agreement is lower for the spatial structure categories than for the semantic categories. This means that for any true two-way sensitivity d_2' , our empirical estimate of d_k' will be lower than indicated by the solid curves in Fig. S3, due to this 'noise' in the ground truth, and this underestimation will be more severe for the spatial structure task than for the semantic tasks.

We can account for this by deriving from Anderson et al (2021)[2] measures of inter-observer agreement in the spatial structure (α_{space}), and semantic (α_{sem}) classification tasks. These measures reflect the proportion of times an observer is expected to agree with the majority view (i.e., mode response) of an image's category, given unlimited viewing time. The derived values are $\alpha_{space} = 0.7154$ and $\alpha_{sem} = 0.8229$. Note that these measures derive from a finite sample of 20 observers and we neglect uncertainty in how the majority opinion might shift with a larger sample.

The deviation in individual observers' conception of the ground-truth category can be modelled in our Monte Carlo simulation by randomly switching the ground-truth category with probability $1 - \alpha$. The resulting corrected relationships between d_k' and d_2' for spatial and semantic categorization are shown as dotted curves in Figure S3. We find that the difference in the reliability of ground truth for spatial structure and semantic tasks has a profound effect: for any value of d_2' the value of d_k' is substantially higher for the semantic task than for the spatial structure task (see dotted lines, Figure S3).

We can use these results to map from d_k' to d_2' for both spatial and semantic tasks, thus correcting for differences in the number of categories *k*, the distribution of stimuli over these categories, and the reliability of ground truth α.

Alternative metrics of task performance, related to Figures S4 and S5

Mutual information quantifies the amount of information shared between two random variables. Let $x \in C$ represent the ground-truth scene category of an image, and let $y \in C$ represent the category assigned by a human participant in the experimental task. The mutual information $I(x, y)$ in bits between x and y is given by

(7)
$$I(x,y) = \sum_{x \in C} \sum_{y \in C} p(x,y) \, log_2 \frac{p(x,y)}{p(x)p(y)}$$

The information in the ground truth category variable is given by $log_2(k)$, where k is the number of ground-truth categories, and the proportion of this information captured by the observer is given by $\frac{I(x,y)}{log_2(k)}$. We find that using this alternative measure for human performance on our spatial and semantic categorization tasks leads to a similar pattern of results as found in our d' analysis (see Figure S4).

Proportion of mutual information captured for semantic/spatial category discrimination exceeds zero at all presentation times, and the semantic task produces better performance than the spatial structure task (Figure S4A). Note that the superior performance on the semantic task is not eliminated after controlling for inter-observer agreement (Figure S4B).

We can also quantify task performance by estimating the variance in categorization responses explained by the ground-truth (R^2). We fit a multinomial logistic regression to raw categorization data, using a logit link function (see MATLAB *mnrfit* function). Results are presented in Figure S5, and closely replicate the results observed for d'. Altogether, d', mutual information and analysis of variance all indicate that semantic discrimination is better than spatial structure discrimination, and that controlling for inter-observer agreement either attenuates these task differences, or has little effect. Importantly, *spatial structure discrimination does not reliably outperform semantic discrimination on any of the tested metrics*.

Relationship between fixation disparity and scene category responses, related to STAR methods

A naïve Bayes classifier trained to predict image category from fixation disparity achieved a classification rate of 35.46% for the semantic category system, and 51.18% for the spatial structure category system. Although a prior-only classifier that always chooses the most common category outperforms this method for semantic classification (37.36%), this isn't the case for spatial structure classification (where prior-only performance is 35.69%).

To test whether participants were using fixation disparity information to predict spatial structure categories, we measured the decision-variable correlation (DVC) between a naïve Bayes classifier that makes use of the fixation disparity cue, and human responses (see main text for discussion of DVC). We found a small negative correlation between observer responses and model responses (r = -.001), indicating that observers were not using fixation disparity to predict spatial structure category.

Task differences as a function of agreement bin, related to Figure 5

Category discrimination performance as a function of presentation time and task, divided into agreement bins, are presented in Figure S6. Note the absence of a reliable task difference across different levels of agreement. For example, for an inter-observer agreement of .9, spatial structure performance exceeded semantic performance, but for an agreement of 1, semantic performance exceeded spatial structure performance.

Alternative method of equalizing inter-observer agreement between tasks, related to figure 5

Here we test an alternative method of equalizing the distribution of inter-observer agreement: by bootstrap-sampling (with replacement) the same number of trials from the semantic and spatial structure tasks in each bin. Task-related performance differences are minimal in this bootstrapped dataset (Figure S7), and closely resemble the results reported in the manuscript (see Figure 5B).

Additional DVC analyses, related to Figure 6

In the main text, we adapted the decision variable correlation method to a 4AFC and 6AFC task by converting categorical responses to binary incorrect/correct responses.

An alternative way to compute the DVC involves treating a k-AFC task (where k is the number of categories) as a set of 2AFC one-vs-all tasks. Consider, for example, our spatial structure categories: flat, closed off, cluttered, and navigable. First let us compute the DVC for the flat category, by dummy-coding the ground-truth as either flat (1), or not-flat (0, i.e., all the other categories). Model and human responses are then also dummy-coded as either flat (1) or not flat (0). Using these data, the DVC can be computed as described in Sebastian and Geisler[3], which yields two correlation values: one for the flat category, and one for the not-flat categories. Note that, if we apply the same process to the closed off category, some of the not-closed off categories are also members of the not-flat category. This means that our individual DVC estimates will be correlated. Nonetheless, using this method produces results that are highly similar to the method described in the main text (see Figure S8), confirming that the spatial → semantic model is considerably worse at predicting categorization behaviour than the semantic → spatial model.

To empirically confirm that the patterns observed in Figure 6 (in the manuscript) are *not* caused by the small difference in model performance, we computed the median participant sensitivity (d' = 1.54) *across both tasks*, and removed (i) participants in the semantic task who performed better than the median, and (ii) participants in the spatial structure task who performed worse than the median. This way, our simple classification model only uses a subset of the full sample of participants, and this subset is biased to produce better performance in the spatial structure → semantic model (see Figure S9A). DVC results are shown in Figure S9B. We then repeated this analysis but removed (i) participants in the semantic task who performed worse than the median, and (ii) participants in the spatial structure task who performed better than the median. This subset is biased to produce better performance in the semantic → spatial structure model (see Figure S9C). DVC results are shown in Figure S9D. If DVC estimates depend on model performance, Figures S9B and S9D should show different trends. However, in both cases, the semantic → spatial structure model predicted human data better than the spatial structure → semantic model (compare red and green solid lines).

Another relevant difference between the two tasks is that human x human DVCs for semantic categorization are higher, on average, than human x human DVCs for the spatial structure categorization.

This shows that humans are more consistent with one another in the semantic task. Could this also affect the difference between the two model DVCs with human data? Using the same median-split procedure as described above, we (i) computed the average pairwise human x human DVC for each participant, and found the median value across both tasks (r = .19), and then we (ii) removed all participants in the semantic task greater than the median, and removed all participants in the spatial structure task less than the median. Similar to above, this has the effect of biasing the model in the opposite direction, and favouring the spatial structure → semantic model. As expected this sampling procedure caused the spatial → semantic model to outperform the semantic → spatial model in terms of d' (see Figure S10A). However, this had little effect on the DVCs: the semantic → spatial structure model still correlated with human categorization data substantially better than the spatial structure → semantic model (see Figure S10B).

The DVC measures human-model agreement while controlling for chance-variation in agreement associated with performance[3]. Another way to quantify trial-by-trial agreement is Cohen's kappa[4]. Typically used to measure inter-rater reliability, Cohen's kappa (κ) is defined as:

(8)
$$\kappa = \frac{p_o - p_e}{1 - p_e}$$

where p_o is the observed agreement between the model and the human, and p_e is the expected chance-level agreement, given the probability distribution of human and model responses. The numerator, $p_o - p_e$, represents the proportion agreement above chance. Figure S11 shows the agreement of the spatial → semantic and the semantic → spatial models with human judgements using this alternative metric. Note that this metric is reported to generally produce larger kappa values for larger category systems[5]. Even with this bias, which favours the spatial → semantic model, we find that the semantic → spatial model producers better predictions of the human data, both in terms of absolute performance, and relative to the pairwise agreement between observers.

Effect of image repetition, related to STAR methods

To examine the effect of image repetition, we conducted two analyses. In our first analysis, we simply measure the improvement in performance when images are presented for the first versus second time. Average response accuracy improved when the same Image appeared for a second time, but this effect was small and only significant in the spatial task (mean spatial structure accuracy improvement = 2.82%, t (29) = -3.7948, p <.001; mean semantic accuracy improvement = 1.11%, t (34) = -1.6567, p = .1068).

However, computing the accuracy difference this way does not control for the confounding practice effect (whereby participants improve as the study progresses). To disentangle the repetition and practice effects, we adopted the following strategy: (i) we divide the human responses into two separate

lists: trials in which images appeared for the first time, and trials in which images appeared for the second time. (ii) We sort the lists by trial order, such that the ‘first-time’ list is in descending order (from the latest trials to the earliest trials), and the ‘second-time’ list is in ascending order. (iii) Working through both sorted lists, we sample images until the mean trial number of both lists converge (with as little error as possible). These two samples contain images that were presented, on average, at approximately the same trial in the experiment. However, one sample contains images presented for the first time, and the other contains images presented for the second time. The accuracy difference between these samples can be used to isolate the repetition effect. In the spatial and the semantic task, the difference between these two groups was non-significant (mean spatial structure accuracy improvement = .60%, t (29) = -.8145, p = .4220; mean semantic accuracy improvement = .95%, t (34) = 1.13, p = .2655). Thus, when we controlled for the practice effect, there was no overall significant effect of repetition on performance.

Supplemental References

1. Hansen, B.C., and Loschky, L.C. (2013). The contribution of amplitude and phase spectra-defined scene statistics to the masking of rapid scene categorization. Journal of Vision *13*, 21-21.
2. Anderson, M.D., Graf, E.W., Elder, J.H., Ehinger, K.A., and Adams, W.J. (2021). Category systems for real-world scenes. Journal of vision *21*, 8-8.
3. Sebastian, S., and Geisler, W.S. (2018). Decision-variable correlation. Journal of vision *18*, 3-3.
4. Cohen, J. (1960). A coefficient of agreement for nominal scales. Educational and psychological measurement *20*, 37-46.
5. Sim, J., and Wright, C.C. (2005). The kappa statistic in reliability studies: use, interpretation, and sample size requirements. Physical therapy *85*, 257-268.
